# Repression of arterial genes in hemogenic endothelium is sufficient for haematopoietic fate acquisition

**DOI:** 10.1038/ncomms8739

**Published:** 2015-07-23

**Authors:** Carlos O. Lizama, John S. Hawkins, Christopher E. Schmitt, Frank L. Bos, Joan P. Zape, Kelly M. Cautivo, Hugo Borges Pinto, Alexander M. Rhyner, Hui Yu, Mary E. Donohoe, Joshua D. Wythe, Ann C. Zovein

**Affiliations:** 1 Cardiovascular Research Institute, University of California, San Francisco, San Francisco, California 94158, USA.; 2 Department of Nutrition, Diabetes, and Metabolism, School of Medicine, Pontificia Universidad Católica de Chile, Santiago 8331150, Chile.; 3 Burke Medical Research Institute, White Plains, New York 10605, USA.; 4 Department of Neuroscience, Brain and Mind Research Institute, Weill Cornell Medical College, New York, New York 10065, USA.; 5 Department of Cell and Developmental Biology, Weill Cornell Medical College, New York, New York 10065, USA.; 6 Department of Molecular Physiology and Biophysics, Baylor College of Medicine, Houston, Texas 77030, USA.; 7 Cardiovascular Research Institute, Baylor College of Medicine, Houston, Texas 77030, USA.; 8 Department of Pediatrics, Division of Neonatology, School of Medicine, University of California, San Francisco, San Francisco, California 94143, USA.

## Abstract

Changes in cell fate and identity are essential for endothelial-to-haematopoietic transition (EHT), an embryonic process that generates the first adult populations of haematopoietic stem cells (HSCs) from hemogenic endothelial cells. Dissecting EHT regulation is a critical step towards the production of *in vitro* derived HSCs. Yet, we do not know how distinct endothelial and haematopoietic fates are parsed during the transition. Here we show that genes required for arterial identity function later to repress haematopoietic fate. Tissue-specific, temporally controlled, genetic loss of arterial genes (*Sox17* and *Notch1*) during EHT results in increased production of haematopoietic cells due to loss of Sox17-mediated repression of haematopoietic transcription factors (*Runx1* and *Gata2*). However, the increase in EHT can be abrogated by increased Notch signalling. These findings demonstrate that the endothelial haematopoietic fate switch is actively repressed in a population of endothelial cells, and that derepression of these programs augments haematopoietic output.

## Abstract (web summary)

The first haematopoietic stem and progenitor cells arise from the hemogenic endothelium of arterial vascular beds. Here the authors describe the mechanism that regulates the endothelial-to-haematopoietic transition and show that *Sox17* and *Notch1*, genes critical to arterial endothelium identity, are also crucial repressors of haematopoietic fate.

## Graphical abstract

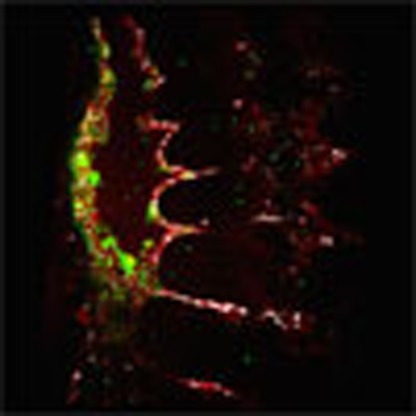

The first haematopoietic stem cells (HSCs) emerge in the embryo from a specialized subset of endothelial cells (ECs), collectively termed as hemogenic endothelium (HE). The concept of endothelial-derived HSCs has broad clinical implications as it may open new avenues for *in vitro* blood production. However, the hemogenic capacity of the endothelium is transient and its precise regulation remains unknown. During a narrow developmental time period (approximately embryonic day (E)10–12 in the mouse^1^,^2^, and 4–6 weeks in the human^3^), hemogenic ECs acquire cell morphology and gene expression consistent with haematopoietic identity, in a process called endothelial-to-haematopoietic transition (EHT)^4^,^5^,^6^. In the mammalian system, the ‘hemogenic window’ is short lived and typified by groups (or clusters) of rounded cells that are observed within the vascular wall. The haematopoietic cell clusters have been demonstrated to contain both haematopoietic stem and progenitor cells (HSPCs)^7^,^8^. Regions known to harbour HE include the aorta-gonado-mesonephros (AGM) region^1^,^9^,^10^,^11^,^12^, vitelline and umbilical arteries^9^,^13^,^14^, yolk sac^15^,^16^, placenta^17^,^18^ and others^19^,^20^, but generally encompass arterial vascular beds, as opposed to the veins or capillaries^21^.

Interestingly, regulators of arterial fate, including the transcription factor Sox17 (ref. 22) and Notch1 (ref. 23), are implicated in haematopoietic emergence from HE, as early loss of either results in haematopoietic defects^24^,^25^. Sox17 positively regulates Notch1 for both arterial fate acquisition and hemogenic endothelial specification^22^,^26^. How these arterial fate specifiers function in endothelial to haematopoietic conversion, separate from their role in artery–vein specification, is unclear.

Here we present data that demonstrates after artery–vein specification, Sox17 actively prevents the transition to haematopoietic fate by repression of key haematopoietic transcription factors, thereby maintaining endothelial identity. The loss of Sox17 promotes haematopoietic conversion, and its dynamic expression imparts a previously unappreciated, but critical step, in endothelial to haematopoietic cell fate transition.

## Results

### Haematopoietic clusters and endothelial gene expression

**Figure 1:**
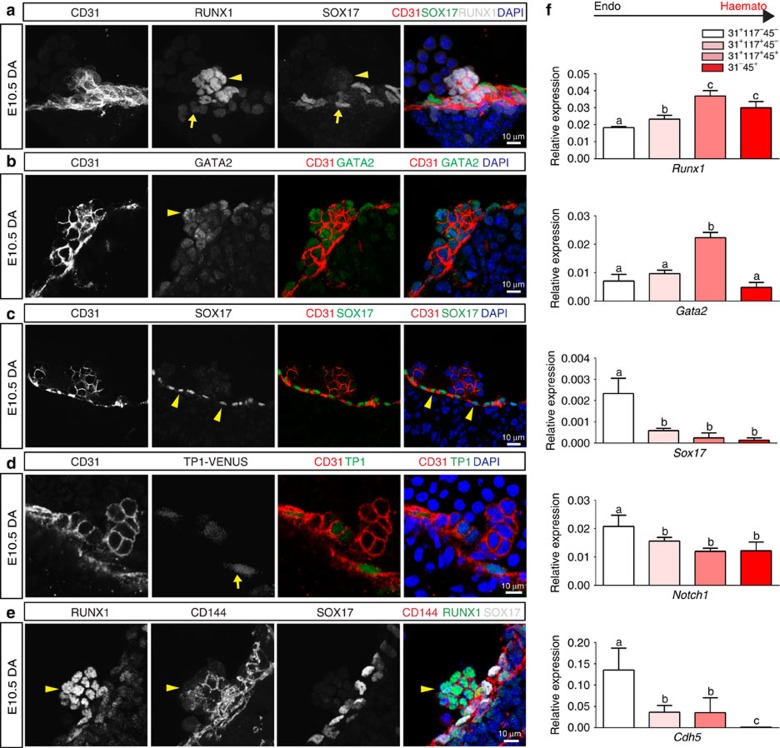
Haematopoietic cell clusters downregulate arterial gene expression. (**a**–**e**) Single channels in black and white, scale bars as shown. E10.5 wild-type dorsal aorta (DA). (**a**) Haematopoietic cell clusters of the AGM at E10.5. The endothelial layer and attached haematopoietic cell clusters are CD31^+^ (red). RUNX1 (grey) is notable in cells comprising the haematopoietic cluster (arrowhead). SOX17 (green) expression is localized to the endothelial layer (arrow). DAPI in blue. (**b**) GATA2 (green) is notable in the haematopoietic cell cluster (arrowhead). CD31 (red) and DAPI (blue). (**c**) SOX17 (green) immunofluorescence is noted in the cell nuclei of the endothelial layer (arrowheads), as compared with the associated cell cluster. CD31 in red, and DAPI in blue. (**d**) Notch pathway activation (green) as measured in the TP1 Venus mouse line is notable in the endothelial layer (arrow) but less so in the associated haematopoietic cell cluster, CD31 in red. DAPI in blue. (**e**) CD144 (red) labels the endothelium and haematopoietic cluster cells (arrowhead), Sox17 in grey, and Runx1 in green. (**f**) Embryos at E10.5 were sorted based on cell surface markers to isolate endothelial cells (CD31^+^CD117^**−**^CD45^**−**^), haematopoietic cluster cells (CD31^+^CD117^+^CD45^**−**^), maturing cluster cells and HSPCs (CD31^+^CD117^+^CD45^+^) and mature haematopoietic cells (CD31^**−**^CD45^+^). Bar graphs depict transcript expression (RT–PCR) in each subgroup for *Runx1*, *Gata2*, *Sox17, Notch1* and *Cdh5* (CD144). Differing letters represent significance between groups where a versus b, or b versus c, or a versus c, is significant to a *P* value<0.01 or less, *n*=3 litters, 24 embryos.

We first evaluated the expression patterns of Sox17, Notch1, Runx1 and Gata2 in the embryonic dorsal aorta (AGM) as all four factors are shown to be required for HSC emergence. The endothelium of this region can be identified by immunofluorescence of the pan-EC surface marker PECAM-1 (CD31), and HSPC clusters are easily apparent through their rounded morphology and shared endothelial marker expression (Fig. 1a–d). RUNX1 (ref. 27) and GATA2 (ref. 28), two transcription factors known to be required for HSPC emergence from HE, are localized to HSPC clusters, as compared with the adjacent endothelium (Fig. 1a,b,e). When known regulators of the arterial program including Notch signalling^23^,^29^ (visualized by the TP1-Venus reporter mouse line^30^,^31^) and SOX17 (ref. 22) are evaluated, immunofluorescence is localized to the endothelium and not the HSPC clusters (Fig. 1c–e). The appearance of HSPC clusters along the aortic wall is coincident with changes in cell surface marker expression, as cluster cells acquire c-Kit (CD117)^7^,^32^,^33^ and CD41 (refs 34, 35) markers (Supplementary Fig. 1a,b), in addition to maintaining endothelial markers CD31 and VE-cadherin (CD144)^36^ (Fig. 1a–e). Eventually, HSPCs also acquire CD45, a pan-haematopoietic surface marker (Supplementary Fig. 1c). Sox17 expression is largely undetectable in cluster cells, but rarely can be seen in a perinuclear pattern with co-expression of Golgi markers (Supplementary Fig. 2d–f), suggesting that it no longer functions as a transcription factor in the cluster cell population. As arterial markers can be flow sensitive^37^,^38^,^39^, we also evaluated the expression patterns of SOX17 and RUNX1 in *Mcl2a*^−/−^ circulation mutants^40^ (Supplementary Fig. 1g), and found that the segregation of SOX17 immunofluorescence to the endothelium and RUNX1 to haematopoietic cell clusters is preserved. The differential expression of surface markers allows for separation of endothelial and haematopoietic populations, as well as HSPC clusters (CD31^+^CD117^+^), by fluorescent-activated cell sorting (FACS) (Supplementary Fig. 1h,i). Transcriptional analyses of sorted populations demonstrate that endothelial subsets (CD31^**+**^CD117^**−**^CD45^**−**^) exhibit lower *Runx1* and *Gata2* transcript levels when compared with HSPC cluster populations (CD31^**+**^CD117^**+**^CD45^**−**^), or as compared with differentiated haematopoietic cells (CD31^**−**^CD45^**+**^) (Fig. 1f; Supplementary Table 1). In contrast, genes associated with arterial identity (*Sox17* and *Notch1*) are decreased in HSPC clusters as compared with the endothelium. Sox17 (refs 26, 41) and Notch1 (refs 24, 42, 43, 44, 45) are known to be important for hemogenic endothelial specification. Thus, the finding that their transcripts and protein levels are actually decreased in HSPC clusters is intriguing. As relatively small populations of primordial germ cells can express CD31 and CD117 (refs 32, 46), we also evaluated populations based on CD41 expression and found that the same trend is observed when we identify hemogenic cluster cells with the marker CD41 (refs 34, 35) (Supplementary Fig. 1b,i–j). Together the data suggest that endothelial to haematopoietic fate conversion may require downregulation of critical arterial genes.

### Sox17 negatively regulates haematopoietic fate

**Figure 2:**
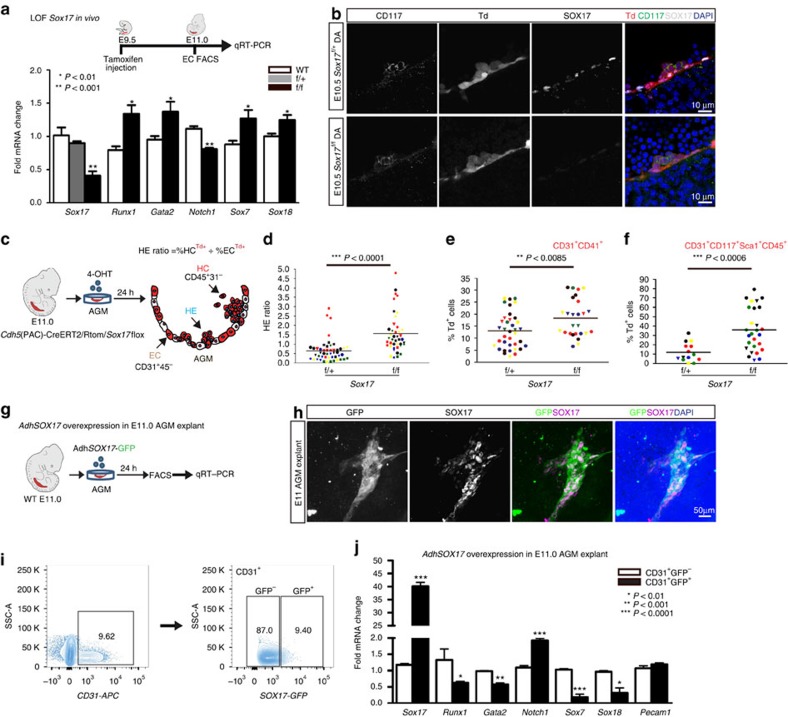
Endothelial to haematopoietic conversion is increased after *Sox17* loss. (**a**) Schema and bar graph of qRT–PCR analyses of sorted endothelial cells from E11 embryos after *in vivo Sox17* ablation at E9.5. Error bars indicate standard error of the mean (*n*=3 litters, embryos pooled by genotype). LOF, loss of function. (**b**) Immunofluorescence of *Sox17* heterozygous and homozygous embryos at E10.5 after *in vivo* Cre induction (tamoxifen induction at E9.5). Haematopoietic clusters are labelled by CD117 (green), Cre traced endothelial and cluster cells in red (Td^+^). SOX17 (grey) is absent in homozygous mutant endothelium. DAPI in blue. DA, dorsal aorta. Scale bar, 10 μm. Single channels in black and white. (**c**) Schematic of AGM explant analysis depicts *in vitro* Cre lineage tracing and calculation of hemogenic output (HE ratio); the ratio between per cent labelled (Td^+^) haematopoietic cells (CD45^+^CD31^**−**^) to per cent labelled (Td^+^) endothelial cells (CD31^+^CD45^**−**^). 4OHT, 4-hydroxytamoxifen. (**d**–**f**) Each data point represents a separate embryo/AGM explant, littermates are depicted by the same data point colour and shape. Bar indicates group mean. *P* values calculated on Student’s *t*-test between groups, significance also validated by two-way analysis of variance, (Supplementary Table 2). (**d**) The HE ratio of *Sox17* homozygous (f/f) and heterozygous (f/+) mutant explants. f/+ *n*=45, f/f *n*=38, 15 litters. (**e**) Percentage of traced Td^+^ hemogenic endothelial cluster cells, designated as CD31^+^CD41^+^. f/+ *n*=37, f/f *n*=26, 9 litters. (**f**) Percentage of traced (Td^+^) maturing HSPCs (identified as CD31^+^CD117^+^Sca1^+^CD45^+^), f/+ *n*=14, f/f *n*=27, 7 litters. (**g**) Schema depicts overexpression analyses in wild-type AGM explants at E11.0. (**h**) Immunofluorescence of E11 AGM explant after human adenoviral SOX17-GFP infection. GFP in green, SOX17 in magenta and DAPI in blue. Scale bar as indicated. (**i**) Cell sorting strategy for endothelial cells (CD31^+^) after exposure to AdhSOX17-GFP (GFP), where GFP^+^ and GFP^**−**^ populations were gated. (**j**) Bar graph of qRT–PCR analyses of sorted E11 AGM CD31^+^ cells after AdhSOX17-GFP infection. Error bars indicate s.e.m. CD31^+^GFP^**−**^ population served as a control, set to one for comparisons of fold change, *n*=3 litters, embryos pooled, *P* values as indicated. (**a**,**j**) *P* values reflect Student’s *t*-test.

To evaluate the impact of Sox17 on EHT, we undertook both loss- and gain-of-function approaches. *In vivo* endothelial genetic deletion of *Sox17* during EHT (induction at E9.5, evaluation at E11; Fig. 2a) was evaluated using a endothelial-specific Cre recombinase (*Cdh5*(PAC)-CreERT2 (ref. 47)) mouse line crossed to a *Sox17* floxed line^25^ with a ROSA26Cre reporter^48^ (RTom, tdtomato, Td^+^). The induction strategy is similar to that used in fate-tracing studies^49^ and allows for timing of *Sox17* endothelial recombination early in the hemogenic window and during EHT. Transcript analysis of sorted ECs after *in vivo* induction uncovered a significant increase in *Runx1* and *Gata2*, two haematopoietic transcription factors known to be critical for HSC development during EHT^27^,^28^,^50^ (Fig. 2a). *Notch1* transcripts are also notably decreased (Fig. 2a), in agreement with previous studies that show Sox17 positively regulates the Notch pathway^22^,^26^. In addition, other members of the SoxF family (*Sox7* and *Sox18*) were increased, possibly due to a compensatory response (Fig. 2a). There were no observed differences in endothelial labelling or cell number across homozygous *Sox17*^*f/f*^, heterozygous *Sox17*^*f/+*^, or control animals (Supplementary Fig. 2a). Immunohistochemical analysis demonstrates the presence of HSPC clusters in the aorta with a marked decrease of endothelial SOX17 in Sox17^*f/f*^ mutants (Fig. 2b). Also, we did not observe any obvious changes in endothelial morphology as evaluated by scanning electron microscopy (Supplementary Fig. 2b).

Currently, it is not possible to predict which specific EC within a hemogenic vascular bed will transition to a haematopoietic fate. Also, not known is whether ECs comprising the same hemogenic site are all capable of EHT. So whether the actual cell fate conversion is a stochastic event or a predetermined fate change remains to be seen. To circumvent the current obstacles of EHT prediction, we adopted a fate-tracing strategy^49^ that allows measurement of traced haematopoietic cell populations from labelled endothelial precursors within a specific hemogenic vascular site. By inducing endothelial recombination of *Sox17* in AGM explants using the *Cdh5(*PAC)-CreERT2/RTom/*Sox17*flox-transgenic mouse line, the number of EHT-derived haematopoietic cells can be quantified by fate mapping (Fig. 2c; Supplementary Fig. 2c; Supplementary Table 2). Tamoxifen induction *in vitro* with the active metabolite 4-hydroxytamoxifen at E11.0 allows immediate ablation in AGM explants during EHT, and the calculation of a HE ratio, which we define as traced haematopoietic cells (HCs) compared with traced ECs. Using this assay to temporally and conditionally ablate *Sox17*, we demonstrate that timed loss of endothelial *Sox17* promotes conversion to haematopoietic cell fate *in situ* (Fig. 2c–f). *Sox17*^*f/f*^ mutants exhibit a significant threefold increase in HE ratios indicating increased haematopoietic output, in addition to significantly increased labelled hemogenic cluster populations (CD31^+^CD41^+^Td^+^) and maturing HSPC populations (CD31^+^CD117^+^Sca1^+^CD45^+^Td^+^; Fig. 2c–f). The observed increase in HE ratios and HSPC number is not due to proliferation effects (as measured by BrdU incorporation; Supplementary Fig. 2d) nor is the higher HE ratio due to changes in cell death (Annexin-V staining; Supplementary Fig. 2d). We also observe increases in other haematopoietic populations (CD31^**−**^CD117^+^Sca1^+^CD45^+^Td^+^; Supplementary Fig. 2e). In addition, when a similar strategy is applied to earlier explants (E9.5) before haematopoietic cell cluster emergence, we observe similar trends in the HE ratio (Supplementary Fig. 2f). So while *Sox17* has been shown to be critical for HE specification before EHT, the loss of *Sox17* actually promotes haematopoietic fate over endothelial fate during EHT. To further evaluate the role of *Sox17* in this process, we undertook gain-of-function studies in wild-type AGM explants using adenoviral-mediated overexpression of human *SOX17* (Adh*SOX17*-GFP; Fig. 2g). Green fluorescent protein (GFP) expression in explants overlapped with SOX17 co-staining (Fig. 2h), allowing for cell sorting of AGM ECs (CD31^+^) that were either successfully infected (GFP^+^) or not infected (GFP^**−**^) by Adh*SOX17*-GFP (Fig. 2i). Transcript analysis of ECs with *SOX17* overexpression demonstrates significant increases in *Sox17* and *Notch1* transcripts with significant reduction in *Runx1*, *Gata2*, *Sox7* and *Sox18* transcripts (Fig. 2j). The data altogether suggest that Sox17 negatively regulates haematopoietic fate through repression of *Runx1* and *Gata2*. We also show the known positive regulation of *Notch1* by Sox17, and regulation of other SoxF family members, *Sox7* and *Sox18*.

### Sox17 represses Runx1 and Gata2

**Figure 3:**
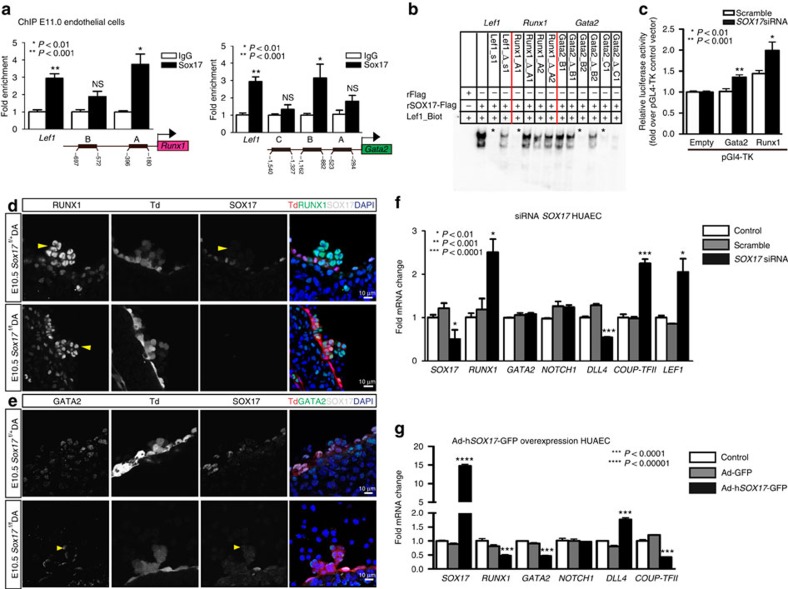
SOX17 directly binds *Runx1* and *Gata2* for repression of haematopoietic fate. (**a**) SOX17 chromatin immunoprecipitation (ChIP) qRT–PCR of E11.0 sorted endothelial cells. Letters denote regions with SOX17-binding site consensus sequences upstream of *Runx1* and *Gata2* promoters, and *Lef1* as a positive control. Error bars indicate s.e.m. IgG control set to one for comparisons of fold change, *n*=3 litters, embryos pooled, *P* values as indicated. (**b**) Electrophoretic mobility shift assay (EMSA) of putative SOX17-binding sites within ChIP sequences designated by letters in **a**. Each lane represents biotin-labelled duplexed oligonucleotides containing the *Lef1* promoter SOX17-binding site (Lef1_Biot). Addition of rSOX17-Flag produces a specific shift, indicating protein–DNA complex (lane 2), which is competed away by unlabelled *Lef1* (Lef1_s1), while mutant probe does not compete (Lef1_Δ_s1). Similar designations are used for putative binding sites (and mutants) in *Runx1* and *Gata2* sequences. Asterisks denote competitive binding. (**c**) Bar graph depicts luciferase activity of *Gata2* and *Runx1* promoters after Sox17 siRNA versus control (scramble). *P* values as indicated. Error bars represent s.e.m. (**d**) Immunofluorescence of haematopoietic cell clusters (arrowhead) in E10.5 dorsal aorta (DA) of *Sox17*^f/f^ and *Sox17*^f/+^ mutants (after tamoxifen mediated Cre induction at E9.5). Traced cells labelled in red (Td^+^), SOX17 in grey and RUNX1 in green. DAPI in blue. Scale bar, 10 μm. Single channels in black and white. (**e**) GATA2 (green) and SOX17 (grey) immunofluorescence of haematopoietic cell clusters in E10.5 DA of *Sox17*^f/+^ and *Sox17*^f/f^ (arrowhead) mutants (Cre induction at E9.5). Traced cells in red (Td^+^), SOX17 in grey and RUNX1 in green. DAPI in blue. Scale bar, 10 μm. Single channels in black and white. (**f**) *SOX17* siRNA knockdown in HUAECs and qRT–PCR analysis. Control represents treatment with lipofectin alone, SOX17 siRNA compared with scrambled (*n*=3 experiments, error bars indicate s.e.m.). *P* values as indicated. (**g**) Adenoviral-mediated overexpression of h*SOX17* in HUAECs and qRT–PCR analyses, *P* values calculated with respect to Adeno-GFP-infected cells, control represents uninfected cells (*n*=3 experiments, error bars indicate s.e.m.).

To determine whether the observed changes in *Runx1* and *Gata2* were due to regulation by SOX17, chromatin immunoprecipitation (ChIP) was carried out in sorted ECs at E11 (Fig. 3a), as well as in human umbilical arterial EC lines, HUAECs (Supplementary Fig. 3a; Supplementary Table 3). Two predicted SOX17-binding sites upstream of *Runx1* and *Gata2* 5′-untranslated regions showed significant enrichment (Fig. 3a). To demonstrate whether SOX17 was capable of direct DNA binding of specific sequences *in vitro*, electrophoretic mobility shift assays (EMSAs) were conducted for sites with high species homology between human and mouse (Supplementary Fig. 3b; Supplementary Table 4). Specific areas within ChIP-enriched regions were capable of outcompeting the known SOX17 regulatory site in the *Lef1* promoter^51^ (Fig. 3b). We further analysed the regulation of *Runx1* and *Gata2* using luciferase reporter assays, which demonstrate derepression of both *Runx1* and *Gata2* activity after Sox17 short interfering RNA (siRNA) knockdown (Fig. 3c). *In vivo* loss of *Sox17* demonstrates intact haematopoietic clusters with normal localization of RUNX1 and GATA2 expression (Fig. 3d,e). To investigate how Sox17 may regulate *Runx1* and *Gata2* in mature endothelium in the human system, we conducted *in vitro* gain- and loss-of-function experiments. *SOX17* siRNA inhibition of human umbilical arterial cell lines resulted in significantly elevated *RUNX1* transcripts, at similar levels to the control *LEF1* (ref. 51), a SOX17 repressive target (Fig. 3f). In addition, genes important in arterial and venous identity are altered with decreased arterial gene transcripts (*DLL4*)^52^,^53^ and elevated transcript levels of *COUP-TFII*, an important determinant of venous fate^21^ (Fig. 3f). In contrast, when *SOX17* is overexpressed after adenoviral infection, *RUNX1* and *GATA2* transcript levels are significantly decreased (Fig. 3g). *SOX17* overexpression also altered levels of *DLL4* (increased) and *COUP-TFII* (decreased) (Fig. 3g). The data suggest a novel role of Sox17 as a repressor of haematopoietic fate, while confirming Sox17 as a pro-arterial fate regulator.

### Intersecting roles of Sox17, Runx1 and the Notch pathway

**Figure 4:**
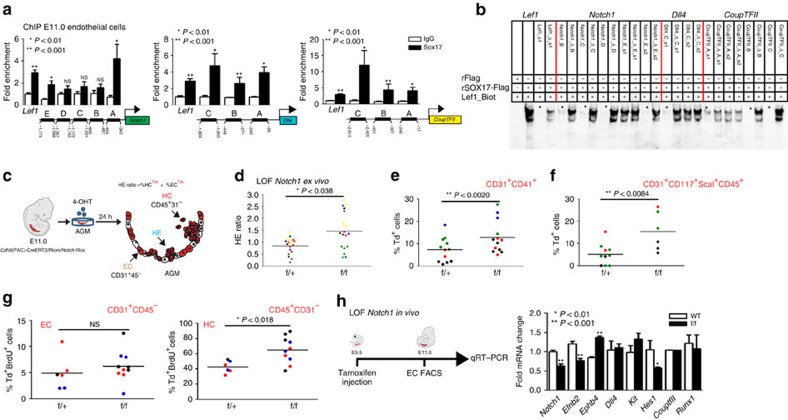
The role of the Notch pathway in endothelial to haematopoietic fate decisions. (**a**) SOX17 (ChIP) qRT–PCR of E11.0 sorted endothelial cells. Letters denote regions upstream of *Notch1*, *Dll4*, and *Coup-TFII* promoters, and *Lef1* as a positive control. Error bars indicate s.e.m.. IgG control set to one for comparisons of fold change, *n*=3 litters, embryos pooled, *P* values as indicated. (**b**) EMSA of putative SOX17-binding sites within ChIP sequences (designated by letters in **a**). Each lane represents biotin-labelled duplexed oligonucleotides spanning the *Lef1* promoter SOX17-binding site (Lef1_Biot). Addition of rSOX17-Flag produces a specific shift, indicating protein–DNA complex (lane 2), which is competed away by unlabelled *Lef1* (Lef1_s1), while mutant probe does not compete (Lef1_Δ_s1). Similar designations are used for putative binding sites (and mutants) in *Notch1*, *Dll4* and *Coup-TFII* sequences. Asterisks denote competition. (**c**) Schematic of AGM explant analysis depicts *in vitro* Cre lineage tracing and calculation of hemogenic output (HE ratio); the ratio between per cent labelled (Td^+^) haematopoietic cells (CD45^+^CD31^**−**^) to per cent labelled (Td^+^) endothelial cells (CD31^+^CD45^**−**^). (**d**–**g**) Each data point represents a separate embryo/AGM explant, littermates are depicted by the same data point colour and shape. Bar indicates group mean. *P* values calculated on Student’s *t*-test between groups, significance also validated by two-way analysis of variance (Supplementary Table 5) (**d**) The HE ratio of *Notch1* homozygous (f/f) and heterozygous (f/+) mutant explants. f/+ *n*=18, f/f *n*=21, 6 litters. (**e**) Percentage of traced Td^+^ hemogenic endothelial cluster cells, designated as CD31^+^CD41^+^. f/+ *n*=12, f/f *n*=13, 4 litters. (**f**) Percentage of traced (Td^+^) maturing HSPCs (identified as CD31^+^CD117^+^Sca1^+^CD45^+^) f/+ *n*=10, f/f *n*=6, 3 litters. (**g**) BrdU^+^ cells measured after 2-h incubation with BrdU in traced ECs (left) and traced HCs (right) demonstrates a significant increase in HC proliferation, f/+ *n*=6, f/f *n*=10, 3 litters. (**h**) Schema and bar graph of qRT–PCR analyses of sorted endothelial cells from E11 embryos after *in vivo Notch1* ablation at E9.5. Error bars indicate s.e.m. (*n*=3 litters, embryos pooled by genotype). LOF, loss of function.

As Sox17 was previously shown to promote arterial identity upstream of the Notch pathway^22^, we evaluated SOX17 regulation of Notch pathway members in our system. SOX17 ChIP demonstrates enriched occupancy upstream of the *Notch1* 5′-untranslated region, and of the Notch ligand *Dll4* (Fig. 4a). In addition, we also observe occupancy upstream of *Coup-TFII*, which has not been previously described (Fig. 4a). Similar enrichment of these sites was observed in HUAECs (Supplementary Fig. 4a). We further validated direct binding of SOX17 within the enriched ChIP sites via EMSA, and demonstrated multiple SOX17-binding sites are capable of outcompeting *Lef1* controls (Fig. 4b; Supplementary Fig. 4b,c). To understand whether *Notch1*, a putative downstream target of SOX17, also plays a repressive role in EHT, we evaluated *Notch1* loss of function. Similar to *Sox17*, loss of *Notch1* in AGM explants increased the HE ratio, as well as populations of HSPCs (Fig. 4c–f; Supplementary Fig. 4d; Supplementary Table 5). We also observed increased HE ratios after AGM explants were exposed the γ-secretase inhibitor DAPT (Supplementary Fig. 4e). However, when BrdU incorporation was evaluated in *Notch1* mutant explants, significantly higher levels of incorporation occurred in the haematopoietic compartment (Fig. 4g), suggesting the observed changes may be due to haematopoietic cell proliferation, and not due to an increase in EHT. Annexin-V levels were not notably changed (Supplementary Fig. 4f,g). *In vivo* loss of *Notch1* (induction at E9.5) demonstrates expected changes in arterial and venous identity genes (*EfnB2* and *EphB4*)^23^ within sorted ECs (Fig. 4h). No changes in *Runx1* transcripts were noted, while expectedly *Hes1* transcripts were decreased (Fig. 4h). There were no observed differences in endothelial labelling or cell number across homozygous *Notch1*^*f/f*^, heterozygous *Notch1*^*f/+*^ or control animals (Supplementary Fig. 4h). Interestingly, we also noted expected changes in endothelial morphology^54^ (Supplementary Fig. 4i).

**Figure 5:**
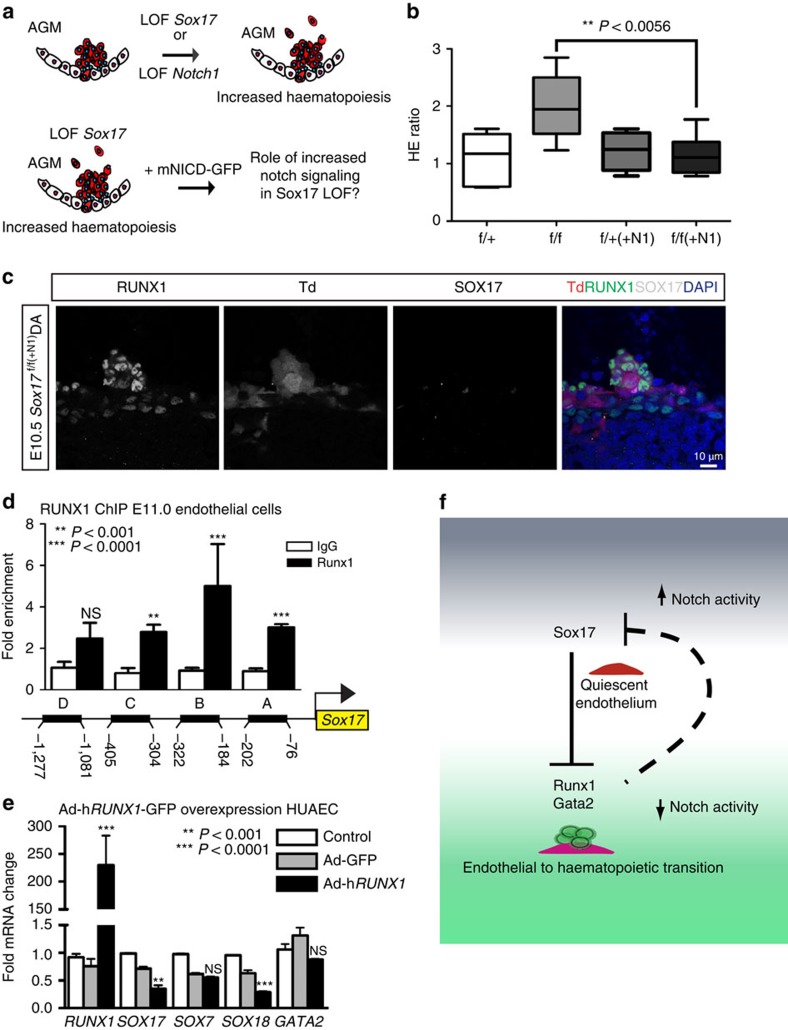
Parsing endothelial and haematopoietic fates during EHT. (**a**) Schematic depicting *Sox17* or *Notch1* loss of function (LOF) and strategy for evaluating Notch overexpression (mNICD-GFP) in *Sox17* mutants. NICD, Notch1 intracellular domain. (**b**) HE ratios of E11 AGM explants in *Sox17* mutants with and without Notch overexpression (+N1). Centre lines represent median values, box represents 25th–75th percentiles and bars represent minimum and maximum values. f/+(−N1) *n*=7, f/+(+N1) *n*=3, f/f (−N1) *n*=5, f/f (+N1) *n*=8, 3 litters. *P* values calculated on Student’s *t*-test between groups, significance also validated by two-way analysis of variance (Supplementary Table 2) (**c**) Immunofluorescence of a representative haematopoietic cluster in a E10.5 *Sox17*^f/f(+N1)^ AGM after *in vivo* induction of Cre and NICD at E9.5. SOX17 in grey, traced ECs (Td^+^) in red and RUNX1^+^ in green. DAPI in blue. Scale bar, 10 μm. (**d**) RUNX1 chromatin immunoprecipitation (ChIP) PCR of E11.0 sorted endothelial cells. Letters denote evaluated regions containing RUNX1-binding site consensus sequences upstream of the *Sox17* promoter. Error bars indicate s.e.m. IgG control set to one for comparisons of fold change, *n*=3 litters, embryos pooled, *P* values as indicated. (**e**) Adenoviral-mediated overexpression of h*RUNX1* in HUAECs and qRT–PCR analyses, *P* values calculated with respect to Adeno-GFP-infected cells, control represents uninfected cells (*n*=3 experiments, error bars indicate s.e.m.). (**f**) Schematic depicting the cell fate switch from endothelial to haematopoietic fate, and the governing regulatory pathways of EHT. Sox17 inhibition of Runx1 and Gata2 maintains endothelial fate. Loss of Sox17 inhibition in the context of decreased Notch activity promotes haematopoietic fate conversion.

To understand the role of Notch1 signalling in the context of *Sox17* loss, we bred R26RNotch1IC-nEGFP lines^55^ (+mNICD-GFP) that overexpress the Notch1 intracellular domain (NICD) upon Cre activation into our temporal endothelial-specific *Sox17* loss-of-function models (Fig. 5a). Increased Notch activation in E11.0 AGM explants was capable of abrogating the observed EHT increase in *Sox17* mutants (Fig. 5b) with normal appearing HSPC clusters *in vivo* after induction of *Sox17* loss and NICD overexpression at E9.5 (Fig. 5c). Thus, the conversion to haematopoietic fate in HE requires loss of arterial identity programs in addition to derepression of haematopoietic genes by SOX17. While our data have shown the regulation of *Runx1* by SOX17, previous reports suggest that RUNX1 may directly bind and repress *Sox17* (ref. 56). To evaluate whether there may be bidirectional regulation in the endothelium, we performed RUNX1 ChIP of conserved sites upstream of *Sox17* transcriptional start sites and found multiple areas of enrichment (Fig. 5d; Supplementary Fig. 4j,k). Adenoviral overexpression of *RUNX1* (Adh*RUNX1*-GFP) in HUAECs demonstrates decreased *SOX17* and *SOX18* transcripts (Fig. 5e). Overall, the data present a complex regulatory network for the maintenance of EC fate and the conversion to a haematopoietic fate (Fig. 5f). Once haematopoietic fate is achieved, both Sox17 and Notch1 have known roles in haematopoietic cell survival^25^ and lineage differentiation^57^, which is also evident in our haematopoietic colony assay evaluation of mutant haematopoietic cells (Supplementary Fig. 5).

## Discussion

An important obstacle in recapitulating HE in culture for *in vitro* blood production is identification of possible activators and silencers of the hemogenic program. Here we demonstrate important altering requirements for Sox17 and Notch1, which highlights the refinements needed for translational models recapitulating EHT. Previous studies have identified Runx1 (ref. 27), Gata2 (ref. 28), Notch1 (refs 24, 58) and Sox17 (refs 25, 26) as critical for EHT. However, dissecting the contributions of these pathways to vascular development versus the process of haematopoietic emergence from the endothelium has not been previously reported. Notch1 and Sox17 both have important roles in arterial specification^22^,^23^,^43^. As the major vessels that harbour HE are arterial sites^9^,^13^, it may be that arterial identity is a prerequisite to hemogenic endothelial activity. However, hemogenic activity also occurs in yolk sac and placental vascular beds that are not overtly arterial^15^,^16^,^17^,^18^. In addition, recent evidence in human ESC cultures suggest that while hemogenic ECs incorporate into arterial vascular walls, they have differential surface marker expression profiles than arterial cells^59^. There is also evidence that arterial identity can be uncoupled from hemogenic capacity^58^,^60^,^61^. So it may be that hemogenic endothelial specification requires the same pathways mobilized in the acquisition of arterial identity, but not arterial identity *per se*. However, for the direct transition to haematopoietic fate, the expression levels of arterial/hemogenic specifiers need to be reduced. The complex temporal requirements, elucidated here, explains previous data where continued or overexpression of Sox17 was noted to prevent haematopoietic conversion in culture^26^,^62^. In addition, the reciprocal repression of Sox17 by RUNX1 introduces another unique aspect of fate determination where once endothelial Sox17 levels decrease, Runx1 levels can rapidly rise during the fate switch, and together they function as a classical bistable system; similar to those described in mesodermal progenitors^63^. Last, the data also demonstrate that the EHT program can be manipulated for increased haematopoietic output, suggesting that hemogenic EC number may not be a fixed entity. If EHT is not restricted to a fixed number of ECs within a hemogenic vascular compartment, but instead occurs as a more global transient stochastic process of developing endothelium, it allows for the possibility of endothelial expansion for HSC production.

## Methods

### Animal care and use

Animal protocols were conducted in accordance with University of California at San Francisco and Baylor College of Medicine Laboratory Animal Research Committee guidelines. *Cdh5*(PAC)-CreERT2 (Tg(*Cdh5*-cre/ERT2)1Rha) mice^47^, *Notch1*^tm2Rko^ and *Sox17*^tm2Sjm^ floxed lines^25^,^57^, and R26RNotch1IC-nEGFP (*Gt(ROSA)26Sor*^*tm1*(Notch1)Dam^) lines^55^ were crossed with R26RTd Cre reporter lines (*Gt(ROSA)26Sor*^*tm14*(CAG**−**tdTomato)Hze^)^48^. TP1-Venus (Tg(Rbp4*-Venus)#Okn) mice^30^,^31^ were generously provided by RIKEN BioResource Centre. Myosin light chain 2 alpha (*Mlc2a*^*−/−*^) mutant lines were provided by Mary Dickinson (Baylor College of Medicine)^40^,^64^. Pregnancies were dated by the presence of a vaginal plug (day 0.5 of gestation). Genomic DNA from adult tail tips or conceptus yolk sacs was genotyped using a MyTaq Extract PCR Kit (Bioline, BIO21127). Genotype PCR was performed using the primers listed in Supplementary Table 6.

### Immunofluorescence and confocal microscopy

E10.5 to E11.5 embryos (*in vivo* induction with maternal tamoxifen injection at E9.5) were fixed in 2% paraformaldehyde solution overnight and frozen in Tissue-Tek OCT Compound (Sakura Finetek, 4583). Cryosections (20–30 μm) were obtained (Thermo Scientific Micron, HM550). Slides were dried for 1 h at room temperature, washed with PBST (0.5% Tween or Triton-X-100) and incubated in blocking buffer (PBST, 1% bovine serum albumin and 5% donkey serum) for 1 h. Primary antibodies (for full list of antibodies please see Supplementary Table 7) were incubated at 4 °C overnight or room temperature for 6 h in blocking buffer. Slides were washed with PBST and incubated with the secondary antibody for 2 h, washed, stained with 2 μg μl^−1^ 4,6-diamidino-2-phenylindole (DAPI) and mounted in Vectashield (H-1400) or Vectamount (Vector Laboratories, H-5000). Images were captured on a Leica SPE Confocal Microscope and compiled using the ImageJ and Imaris 7.6 (Bitplane; Belfast, UK) software.

### Flow cytometric analyses and cell sorting

Whole embryos or AGMs underwent mechanical dissociation by pipetting to single cell suspension in Hank’s Balanced Salt Solution with 2% fetal bovine serum, 1% penicillin/streptomycin and 10 mM HEPES, pH 7.2 (ref. 65) and stained for 30 min at 4 °C with agitation. Single cell suspensions were sorted in a BD FACS Aria III. Flow cytometric analyses were performed on a FACS Verse or FACS Aria III using the FACSDiva 8.0 software (BD Biosciences) and data analysed using the FlowJo v10.0.7 (Tree Star). Gating strategy in Supplementary Figs 1h,i, and 2c, see Supplementary Table 7 for a list of antibodies.

### Real-time RT–PCR expression analysis

For *in vivo* transcriptional characterization of the induced endothelium, lineage traced CD31-APC^+^,CD41-FITC^**−**^,CD45-FITC^**−**^ DAPI-excluded cells were sorted (for full list of antibodies please see Supplementary Table 7) into MCDB-131 complete medium and RNA was immediately extracted using RNeasy Plus Micro Kit (Qiagen, 74034). RNA (50–300 ng) was reverse transcribed using Superscript III Reverse Transcriptase (Life Technologies, 18080044) according to the manufacturer’s instructions and complementary DNA (cDNA) was quantified with Fast SYBR Green Master Mix (Life Technologies, 4385612) in a CFX384 Touch Real-Time PCR Detection System (Bio-Rad). Fluorescence was interpreted relative to *GAPDH* housekeeping gene expression and quantified using the ΔCt method to obtain relative expression or the ΔΔCt method for fold change values, as indicated. A full list of oligonucleotide sequences is listed in Supplementary Table 1.

### AGM explant culture and *in vivo* induction

AGMs from *Cdh5*(PAC)-CreERT2/R26RTd/*Sox17* and *Notch1* floxed embryos were dissected and cultured for 24 h on 40 μm filters at an air liquid interface in 10 μM 4-hydroxytamoxifen (Sigma H7904) in myelocult medium (Stem Cell Technologies) supplemented with 10^**−**6^ M hydrocortisone (Stem Cell Technologies)^49^, at E11 (and E9.5 for *Sox17* mutants). *In vivo* induction was achieved by intraperitoneal injection of 0.8 mg of tamoxifen of pregnant dams at E9.5. Tamoxifen powder (MP Biomedical, 156738) was dissolved in a sunflower seed oil/ethanol (10:1) mixture at 10 mg ml^−1^ (ref. 49). DAPT γ-secretase inhibitor (Sigma, D5942) was prepared in dimethylsulphoxide and added directly to explant culture medium at final concentrations of 25, 50, 100 or 200 μM. For overexpression studies, AGMs were incubated with 8 × 10^7^ adenoviral particles per millilitre at 37 °C with agitation for 1 h before explant culture^49^. Adeno-CMV-hSox17-GFP (AdhSox17-GFP) was produced by Vector Biolabs (ADV-224019, RefSeq: BC140307).

### BrdU

AGM explants were incubated for 2 h with BrdU (10 μM), disaggregated and stained for extracellular markers CD45-percp and CD31-APC for 30 min. Cells were then fixed and permeabilized with BD Cytofix/Cytoperm (BD Biosciences, 554714) according to the manufacturer instructions. Cell pellet was washed and incubated in DNase I (300 μg ml^−1^) for 1 h at 37 °C, stained with DAPI and anti-BrdU conjugated with FITC for 30 min, and analysed by flow cytometry.

### Annexin-V

AGM explants were disaggregated, washed in PBS and resuspended in buffer (10 mM HEPES, 0.9% NaCl, 2.5 mM CaCl_2_ and 0.1% bovine serum albumin) containing FITC-conjuated Annexin-V (BioLegend, 640906). Cells were incubated at room temperature in the dark for 15 min followed by the addition of buffer containing DAPI, and analysed by flow cytometry.

### siRNA

Primary human umbilical arterial ECs (HUAEC) (VEC Technologies) were cultured in MCDB-131 Complete medium (VEC Technologies). *Sox17* Silencer Select siRNA (Ambion, s34626-8), scramble negative control siRNA (non-targeted sequences), versus water (control) was administered using Lipofectin (Invitrogen, 18292011), and RNA was extracted 48 h later using the RNeasy Mini Kit (Qiagen, 74104). Real-time PCR with reverse transcription (RT–PCR) was conducted as described above. All cell culture experiments were carried out between passages 4 and 6. Supplementary Table 1 lists oligonucleotide sequences of Real-time RT–PCR primers.

### Recombinant adenovirus

Recombinant adenoviral particles were produced by Vector Biolabs (Philadelphia, PA, USA). Human SOX17 adenovirus (AdhSOX17-GFP) contains Sox17 cDNA (GenBank RefSeq ID BC140307) and enhanced GFP (eGFP) driven by CMV promoters. Human RUNX1 adenovirus (AdhRUNX1-GFP) contains eGFP-2A preceding RUNX1 cDNA (RefSeq ID BC136381) driven by a single CMV promoter. Ad-GFP control adenovirus (cat# 1060) contains CMV driving eGFP only. Viral particles per cell (1–3 × 10^2^) were used to infect subconfluent HUAECs 36 h before RNA extraction. All cell culture experiments were carried out between passages 4 and 7.

### Chromatin immunoprecipitation

Briefly, HUAEC or E11 CD31-APC^+^ cells were cross-linked with 1% formaldehyde, quenched with 0.125 M glycine and resuspended in lysis buffer (50 mM HEPES-KOH pH 7.5, 140 mM NaCl, 1 mM EDTA, 10% glycerol, 0.5% NP-40, 0.25% triton X-100 in double distilled water) containing protease inhibitors. The chromatin solution was sonicated, and the supernatant diluted 10-fold. An aliquot of total diluted lysate was used for input genomic DNA control. Primary antibody or IgG control was incubated with Pierce Protein A/G Magnetic Beads (Thermo Scientific, 88803) at 4 °C overnight to preclear the sample. Sox17 antibody (R&D Systems, AF1924) was used to ChIP in both sorted ECs and HUAEC samples, while Runx1 antibody (Cell Signalling, D4A6) was used to perform ChIP in HUAECs. The magnetic bead coated by the antibody was washed (PBS, 0.1% Triton X-100) then incubated with the precleared sample at 4 °C overnight. The precipitates were washed, and the chromatin complexes were eluted. After reversal of cross-linking (65 °C for 4 h), the DNA was purified using QIAquick PCR purification kit (Qiagen, 28104) and 100 pg was used as a template in each quantitative PCR reaction for quantitative analysis. Oligonucleotides used in PCR for quantitative ChIP are listed in Supplementary Table 3. Antibody dilutions are listed in Supplementary Table 7.

### Non-radioactive electrophoretic mobility shift assay

Recombinant SOX17-Flag and Flag alone (pcDNA3 vector (Promega)) were expressed in 293T cells. Plasmids were transfected using Lipofectamine 2000 Transfection Reagent (Life Technologies, 11668019) 36 h before cells were lysed in RIPA buffer containing protease inhibitors. Recombinant protein was immunoprecipitated from lysate overnight at 4 °C with Anti-FLAG M2 magnetic beads (Sigma, M8823) and the recombinant protein eluted with excess FLAG peptide. 5–7 μl of the first eluate was used in a binding reaction along with 0.3 pmol of complementary annealed 3′ Biotin-labelled oligonucleotides (Integrated DNA Technologies), 300-fold excess competitor probes, 0.02U Poly(dG–dC) (Sigma, P9389) and binding buffer (100 mM HEPES pH 8.0, 50 mM KCl, 500 μM dithiothreitol, 50 μM EDTA, 1 mM MgCl_2_ and 5% glycerol by volume)^66^. DNA–protein complexes were resolved on 7% native polyacrylamide gel, transferred to neutrally charged nylon membrane, incubated with Streptavidin-POD (Roche, 11089153001) and imaged by chemiluminescence. See Supplementary Table 4 for probe sequences.

### Luciferase reporter assay

Putative regulatory sequences (700–850 bp) including *Sox17* ChIP-enriched regions and EMSA-competent SOX17-binding sites were synthesized and cloned (Integrated DNA Technologies) based on UCSC genome browser murine sequences (see Supplementary Methods for fragment sequences). The fragments were amplified by PCR (Phusion, New England Biolabs) with appropriate linkers. The pGL4-TK vector (pGL4.54, Promega), containing the gene encoding *Firefly* luciferase driven by a TK minimal promoter, was digested using kpnI restriction enzyme (New England Biolabs) and mung bean nuclease (New England Biolabs) followed by ligation using Gibson Assembly Master mix (New England Biolabs) and confirmatory sequencing. C166 murine yolk sac ECs (30,000, ATCC, CRL-2581) were reverse cotransfected with 400 ng of reporter vector along with 10 ng of a *Renilla* luciferase transfection control plasmid (pRL, Promega) and 30 pmol of a Sox17-targeted or non-targeted ‘scramble’ siRNA pool (ON-TARGETplus siRNA SMARTpool, GE Dharmacon) using Lipofectamine 3000 (Life Technologies) according to manufacturer’s recommendations. After 48 h of culture, cells were lysed and luciferase activity assessed using the Dual-Luciferase Reporter Assay System reagents (Promega) in a GloMax 96 Microplate Luminometer with dual injectors. In technical triplicate, relative luciferase activity was calculated by dividing *Firefly* readings by *Renilla* readings for each well and then normalized according to baseline values for each treatment condition after transfection of pGL4-TK without a fragment added.

### Statistical analyses

Student’s *t*-test, one-way and two-way analysis of variance analyses were performed as indicated in all experiments where *n*≥3 unless otherwise noted. Mean and standard error were calculated and graphed using GraphPad Prism 6 software. All statistical measurements are listed in Supplementary Tables 2 and 5.

## Additional information

**How to cite this article:** Lizama, C. O. *et al*. Repression of arterial genes in hemogenic endothelium is sufficient for haematopoietic fate acquisition. *Nat. Commun.* 6:7739 doi: 10.1038/ncomms8739 (2015).

## Supplementary Material

Supplementary Information Supplementary Figures 1-6, Supplementary Tables 1-7, Supplementary Methods, and Supplementary References
