# Supplementary material for: Repression of arterial genes in hemogenic endothelium is sufficient for haematopoietic fate acquisition
Source: Nat Commun. 2015 Jul 23;6:7739. doi: 10.1038/ncomms8739 (PMC4519987; doi:10.1038/ncomms8739)
Supplement: Supplementary Information — Supplementary Figures 1-6, Supplementary Tables 1-7, Supplementary Methods, and Supplementary References [file ncomms8739-s1.pdf]

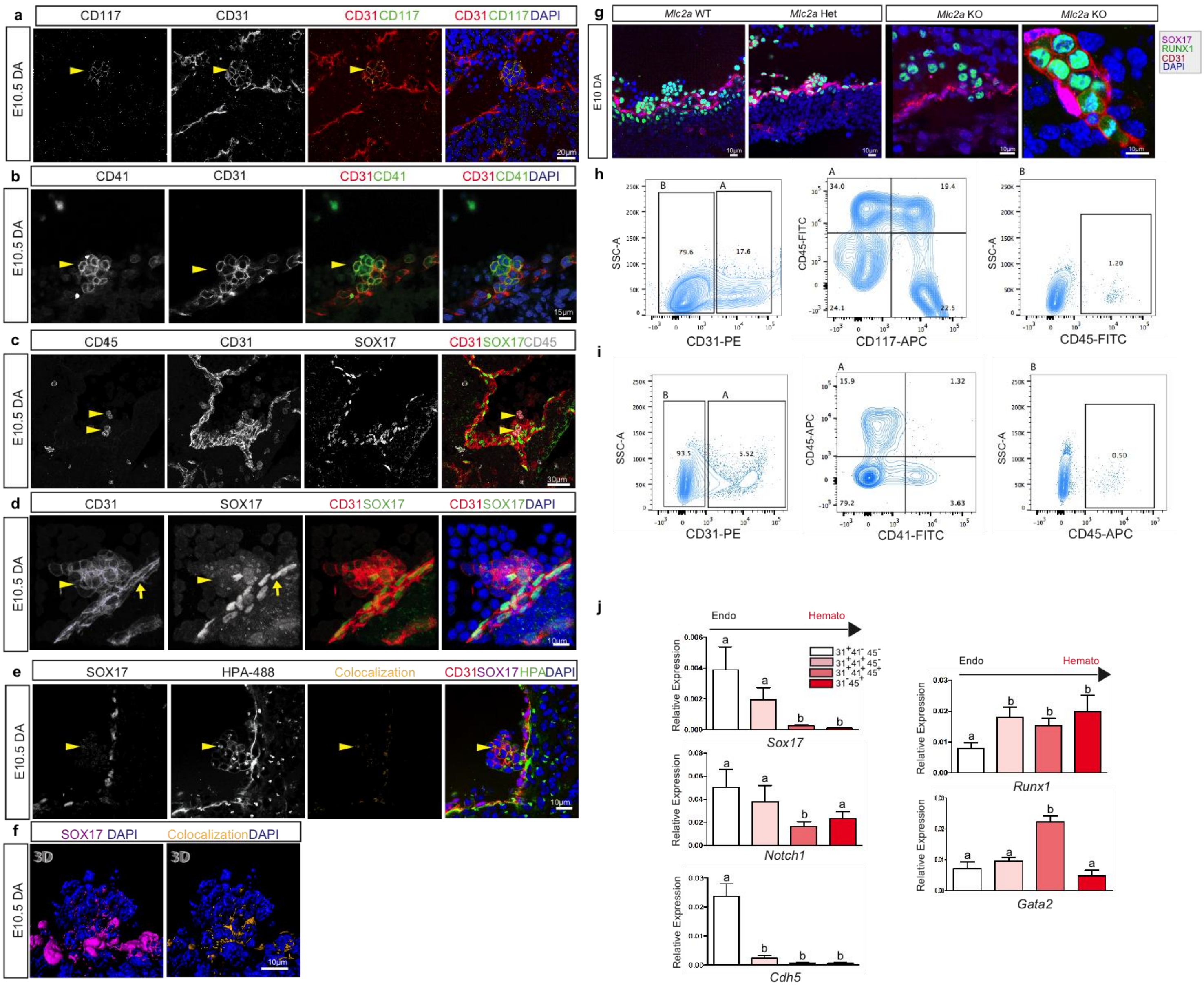

Supplementary Figure 1: Surface marker and gene expression of hematopoietic cell clusters.

(**a**) The endothelial layer and attached hematopoietic cell clusters are CD31$^+$ (red), while CD117$^+$ (green) identifies cells in the HSPC clusters (arrowhead).

(**b**) CD41 marker expression in green is notable in endothelial associated hematopoietic cell clusters (arrowhead) identified by CD31$^+$ staining (red).

(**c**) CD45$^+$ (grey) is noted in mature HCs and few cluster associated cells (arrowheads). SOX17 (green) is localized to the underlying endothelium. CD31$^+$ staining in red.

(**d**) Sox17 (green) strongly marks nuclei of CD31$^+$ (red) endothelial cells (arrow) compared to the dim punctate staining apparent in HSPC clusters (arrowhead).

(**e**) CD31 (red), Sox17 (purple), and fluorescent conjugated Lectin HPA (HPA-488, green), a protein with a strong affinity for the golgi apparatus membranes. Punctate Sox17 staining in the HSPC cluster (arrowhead) co-localizes with the golgi marker (Co-localization in orange).

(**f**) HSPC cluster from (**e**) volume-rendered to highlight Sox17 and HPA-488 co-localization.

(**a**-**f**) DA= dorsal aorta, DAPI nuclear stain in blue, scale bar as stated in μm.
(**g**) *Mlc2a*$^{-/-}$ mutants exhibit disrupted CD31^{+} (red) Sox17^{+} (purple) aortic endothelial architecture, while emerging HSPC clusters appear phenotypically normal in the absence of circulation. Runx1^{+} (green) marks HSPC clusters. KO, knockout is *Mlc2a*$^{-/-}$; Het, heterozygous is *Mlc2a*$^{+/-}$; WT, wildtype. DAPI in blue.
(**h**) Gating strategy for E10.5 wildtype embryonic cell isolation, FACS sorted using CD31, CD117, and CD45 conjugated antibodies prior to transcript analysis.
(**i**) Gating strategy as in (**h**) using markers CD31, CD41, and CD45.
(**j**) E10.5 wildtype embryo cells sorted using CD41^{+} as a marker of HSPC clusters. Enriched populations of endothelial cells (CD31^{+}CD41^{-}CD45^{-}), HSPC cluster cells (CD31^{+}CD41^{+}CD45^{-}), maturing HSPC cluster/HSC cells (CD31^{+}CD41^{+}CD45^{+}), and mature hematopoietic cells (CD31^{-}CD45^{+}) were evaluated for gene expression via Real Time RT-PCR (bar graphs). Real Time RT-PCR demonstrates increased *Runx1* and *Gata2* transcripts in populations transitioning from endothelial cells to hematopoietic cluster cells, and decreased *Sox17, Notch1,* and *Cdh5* transcripts. Differing letters represent significance between groups where a versus b is significant to a p value < 0.01 or less. Error bars indicate standard error of the mean (SEM). n=3 litters.

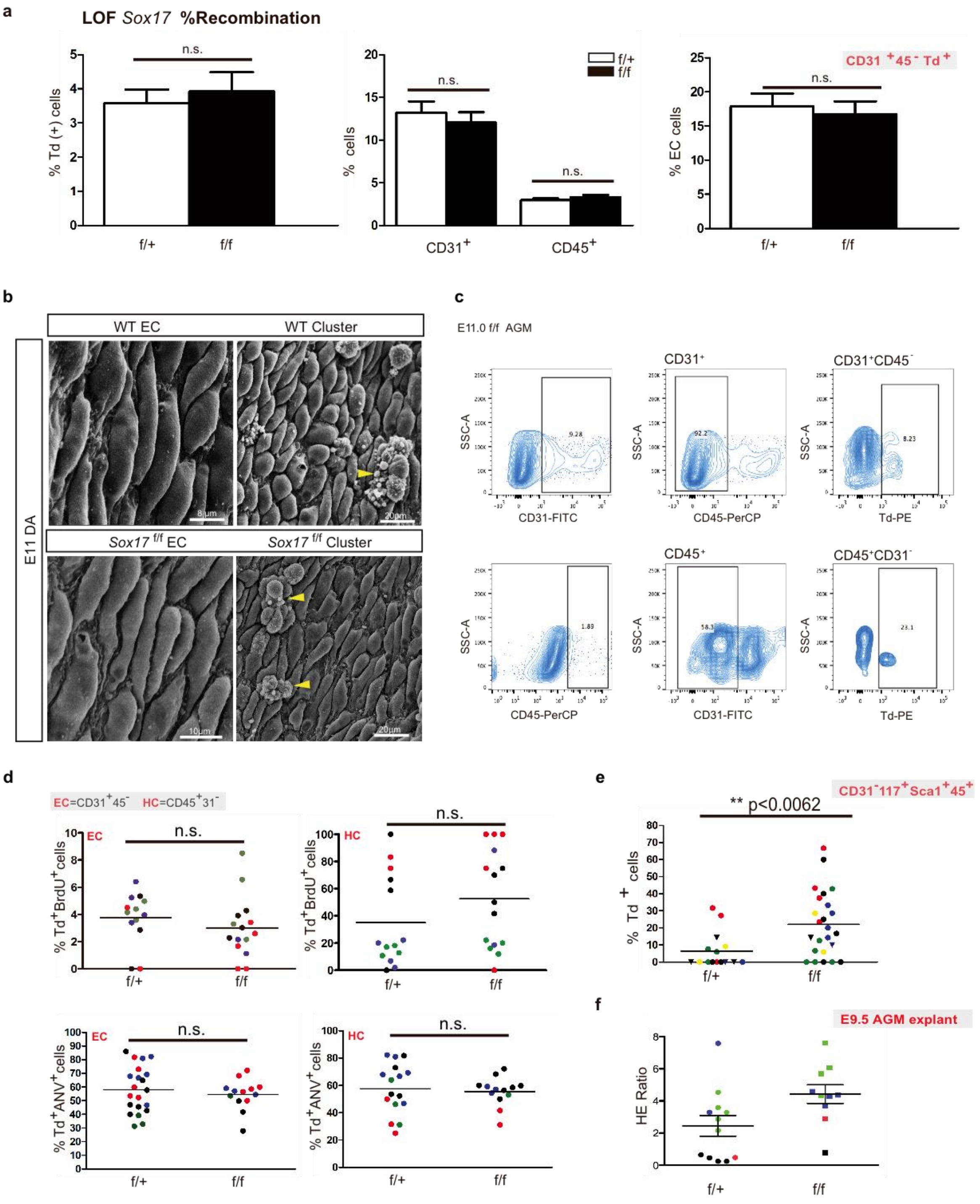

Supplementary Figure 2: Temporal endothelial loss of *Sox17*.

(**a**) Recombination levels of *Sox17*$^{f/f}$ explants as measured by tdTomato (Td^{+}) detection by FACS. Cells from homozygous embryos that express detectable tdTomato are presumed to have recombined at least one R26R allele. Among the compartments analyzed, no significant differences in recombination were found between f/+ and f/f cells. Left most graph depicts total number of cells that were traced (Td+), middle graphs total % of cells within endothelial cell, EC (CD31^{+}) and hematopoietic cell, HC (CD45^{+}) populations (traced and untraced), and rightmost graph is the percent of ECs that were traced (Td^{+}). Error bars indicate SEM. Significance was determined by Student’s t-test. n.s. = not significant.

(**b**) Scanning electron micrographs of wildtype and *in vivo* Cre induced (tamoxifen induction at E9.5) *Sox17*$^{f/f}$ dorsal aortic sections from E11 embryos. Arrowheads indicate endothelial-associated clusters with hematopoietic morphology. There are no appreciable differences in the endothelial layer or associated clusters after *Sox17* ablation. WT, wildtype.
(**c**) Gating strategy for populations evaluated in the calculation of the HE ratio.
(**d**) Evaluation of proliferation and cell death after *Sox17* loss of function. Top: BrdU incorporation after 2 hours of incubation in AGM explants of *Sox17* floxed embryos after tamoxifen Cre induction demonstrates no significant differences in BrdU incorporation of ECs ($Td^{+}CD31^{+}45^{-}$) or HCs ($Td^{+}CD45^{+}CD31^{-}$). (f/+ n=14, f/f n=15). Bottom: Cell death analysis, as measured by AnnexinV+ staining, shows no significant differences in either EC or HC populations (f/+ n=18, f/f n=13).
(**e**) Percentages of traced (Td+) cells of other hematopoietic subsets ($CD31^{-}CD117^{+}Sca1^{+}45^{+}$) are significantly increased in homozygous explants.
(**f**) E9.5 AGMs were explanted and induced as in figure 2c, followed by FACS analysis for determination of the HE ratio 24hrs later (f/+ n=12, f/f n=10). Sox17 homozygous mutant explants trend toward a higher HE ratio compared to heterozygotes, but do not reach significance by t-test, but shows significance in 2-way ANOVA analysis (Supplementary Table 2).
(**d**-**f**) Each data point represents a separate embryo/AGM explant, littermates are depicted by the same data point color and shape. Bar indicates group mean. *P*-values calculated on student's t-test between groups. n.s. = not significant.

a

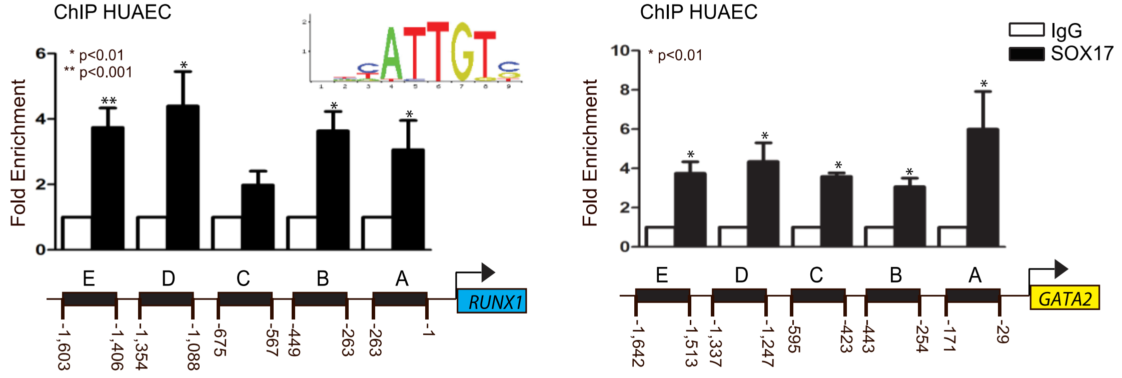

b

Runx1

*Runx1*_A1

Mus musculus	TTGCAGAAACAAATATTCAAATTGTTAAAGATTAAAAAAA
Homo sapiens	TTGTAAAAACAAATATTCAAATTGTTAAAGATTAAAAAAA
Pan troglodytes	TTGTAAAAACAAATATTCAAATTGTTAAAGATTAAAAAAA
Consensus	TTGTAAAAACAAATATTCAAATTGTTAAAGATTAAAAAAA

*Runx1*_A2

Mus musculus	CCTGTGGTTTGCATTCAGTGTGATTCGTCCTGCCTGCTGACCACT
Homo sapiens	CCTGTGGTTTGCATTCAGTGTGATTCGTCCTGCCTGCTGACCACT
Pan troglodytes	CCTGTGGTTTGCATTCAGTGTGATTCGTCCTGCCTGCTGACCACT
Consensus	CCTGTGGTTTGCATTCAGTGTGATTCGTCCTGCCTGCTGACCACT

Gata2

*Gata2*_B1

Mus musculus	GGCGCGCGGCGCTGATTGGCTGGCGCCGGCTTCAT
Homo sapiens	GGCGCGCGGCGTTGATTGGCTGGCTTGGGCTTCTT
Pan troglodytes	GGCGCGCGGCGTTGATTGGCTGGCTTGGGCTTCTT
Consensus	GGCGCGCGGCGTTGATTGGCTGGCTTGGGCTTCTT

*Gata2*_B2

Mus musculus	GCGGGAGCAGCCAATGGGGGGGCGGAGGCTGGGCGG
Homo sapiens	GCGGACGCAGCCAATGGGAGG-CGGAGGCTGGGAGG
Pan troglodytes	GCGGACGCAGCCAATGGGAGG-CGGAGGCTGGGAGG
Consensus	GCGGACGCAGCCAATGGGAGG-CGGAGGCTGGGAGG

Supplementary Figure 3: Conservation of SOX17 regulatory sites in hematopoietic genes across species and cell type.

(**a**) SOX17 ChIP was performed in human cell lines (HUAECs) and putative binding sites of *RUNX1* and *GATA2* were evaluated. Error bars indicate standard error of the mean. Inset left: SOX17 binding site consensus sequence. n=3, *p*-value reflects student's t-test.

(**b**) EMSA validated SOX17 binding sites for *Runx1* and *Gata2* demonstrate evolutionary conservation.

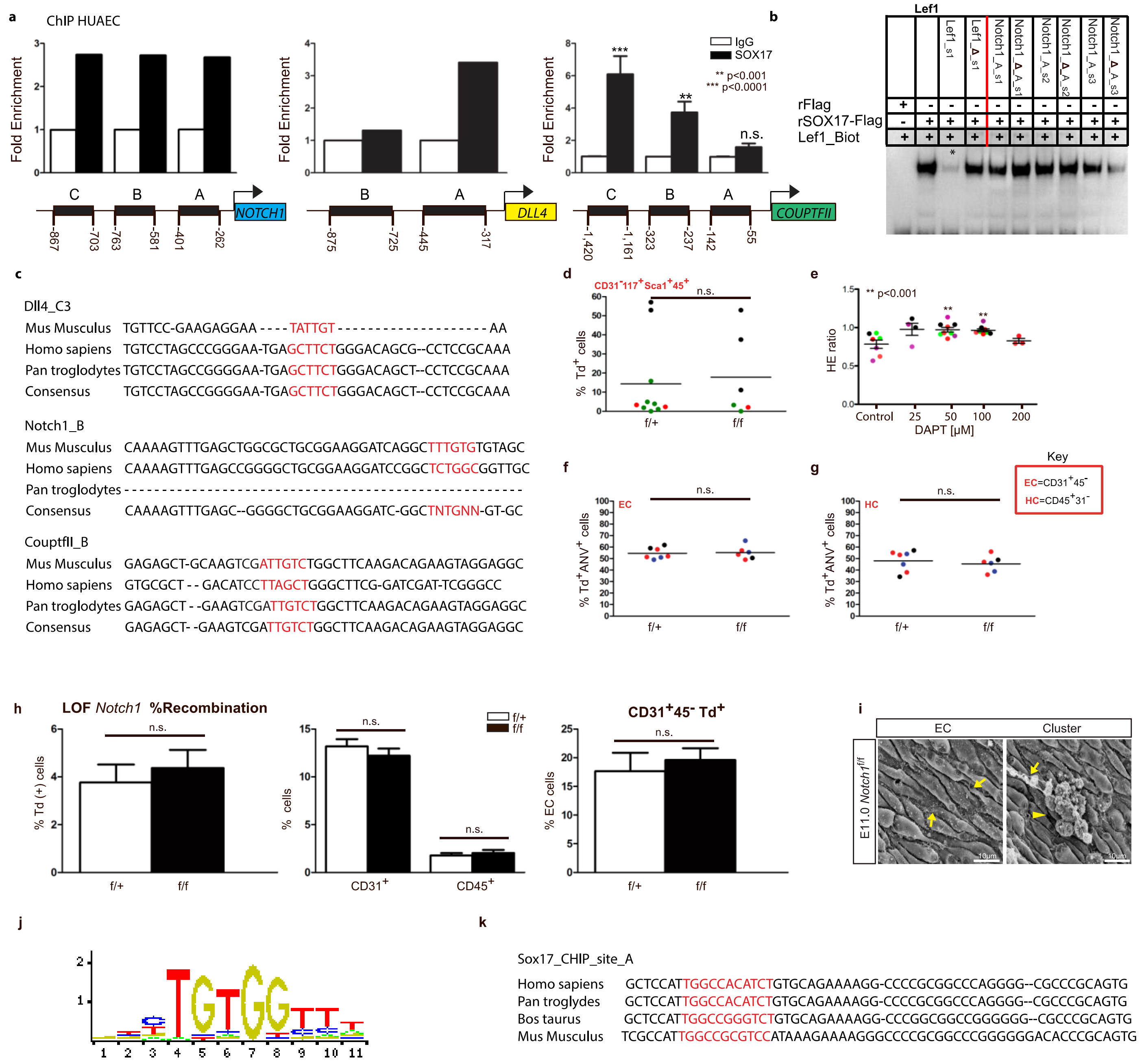

Supplementary Figure 4: Notch pathway targets, and Notch loss of function in EHT.

(**a**) SOX17 ChIP in HUAECs and putative binding sites of *NOTCH1*, *DLL4*, (n=1) and *COUPTFII* (n=3). *P*-values reflect student's t-test.

(**b**) EMSA of murine *Notch1* ChIP site A (figure 4a), which exhibited the highest enrichment, does not demonstrate *in vitro* binding, suggesting possible co-binding partners for this particular ChIP region.

(**c**) Evolutionary conservation of EMSA validated SOX17 binding sites for *Notch1*, *Dll4* and *CoupTFII*.

(**d**) *Notch1* explants evaluated for traced hematopoietic populations ($CD31^{-}CD117^{+}Sca1^{+}45^{+}Td^{+}$). n=3 litters, n.s.= not significant.

(**e**) DAPT treated AGM explants at E11 at the indicated molar concentration. Control explants were treated with DMSO (vehicle). A significant increase in the HE ratio is visible with 50-100 μM DAPT in comparison to control. Error bars indicate SEM. *P*-values based on one-way ANOVA. Control n=7, 25μM n=4, 50μM n=9, 100μM n=8, 200μM n=3.

(**f**) Annexin V^+ staining in the $CD31^+CD45^-Td^+$ traced endothelial cell (EC) populations from *Notch1* AGM explants. f/+ n=7, f/f n=6 from 3 litters.

(**g**) Annexin V^+ staining in hematopoietic cell (HC) $CD45^+CD31^-Td^+$ populations from *Notch1* AGM explants. f/+ n=7, f/f n=6 from 3 litters.

(**d**-**g**) Each data point represents a separate embryo/AGM explant, littermates are depicted by the same data point color and shape. Bar indicates group mean. *P*-values calculated on student's t-test between groups.

(**h**) Recombination levels in *Notch1* AGM explants. No significant differences were found between f/+ and f/f cells. Left most graph depicts total number of cells that were traced (Td^+), middle graph total % of cells within EC ($CD31^+$) and HC ($CD45^+$) compartments (traced and untraced), and rightmost graph is the percent of ECs that were traced (Td^+). Error bars indicate standard error of the mean. Significance was determined by student's t-test. n.s. = not significant.

(**i**) Scanning electron microscopy of *in vivo* Cre induced *Notch1*$^{f/f}$ dorsal aortic sections at E11 (tamoxifen induction at E9.5) demonstrate EC-associated projections (arrows). Hematopoietic clusters appeared to exhibit relatively normal morphology (arrowhead).

(**j**) Runx1 binding site consensus sequence.

(**k**) Sequence and evolutionary conservation of Runx1 ChIP-enriched site A (figure 5d) within the Sox17 promoter.

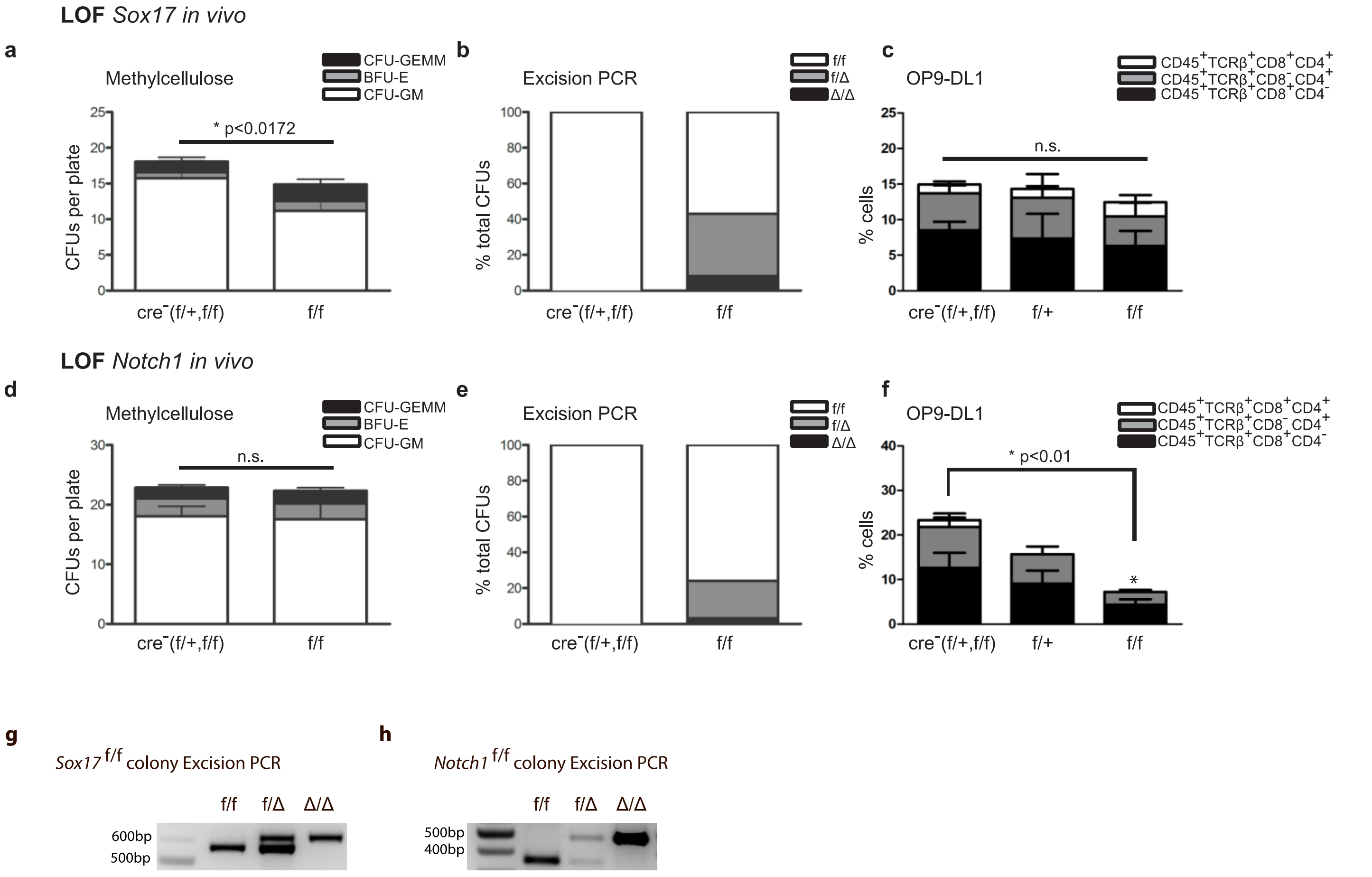

Supplementary Figure 5: *Sox17* and *Notch1* requirements in hematopoietic development.

(**a**) Traced hematopoietic populations ($Td^+CD117^+CD45^+$) from *in vivo* induced $Sox17^{f/f}$ (tamoxifen induction at E9.5) were sorted and plated in methylcellulose CFU assays.

Colony forming units (CFUs) were scored after 7 days as granulocyte, erythrocyte, monocyte, and megakaryocyte (CFU-GEMM), granulocyte and monocyte (CFU-GM), and burst-forming units with erythrocytes (BFU-E). There exists a significant decrease in CFU capacity of *Sox17* homozygous animals. Error bars represent standard error of the mean. *P*-value reflects a student's t-test on total number of colonies. (n=20 embryos from 3 litters)

(**b**) Colonies were genotyped from Cre positive f/f embryos for two excised alleles (Δ/Δ), one excised allele (f/Δ), or no excision of floxed exons (f/f). Percentage of CFUs by genotype. Double excision (Δ/Δ) occurs at similar percentages as to known inducible endothelial Cre recombination measured by Td^+ (n=75 CFUs).

(**c**) The same populations as (**a**) were co-cultured on OP9-DL1 stromal cells in a lymphoid differentiation assay. After 5 week OP9-DL1 co-culture, cells were evaluated for T lymphoid subtypes ($CD45^+TCR□^+$ and CD8 and CD4 single and double positivity). *Sox17* homozygous animals exhibited decreased percentages of all subtypes, but the differences were not significant. Error bars indicate standard error of the mean. (n=19 embryos from 3 litters).

(**d**) CFU assays as described above for $Notch1^{f/f}$ embryos. No significant decrease in $Notch1^{f/f}$ CFU ability is observed (n=25 embryos from 3 litters). n.s.= not significant.

(**e**) There is a relatively low number of fully excised colonies Δ/Δ, suggesting a loss of this cell population (n=233 CFUs).

(**f**) OP9-DL1 co-culture of cells from *Notch1*$^{f/f}$ embryos demonstrates significantly decreased lymphoid differentiation capacity (n=13 embryos from 3 litters). *P*-value reflects student's t-test.
(**g**) Excision of *Sox17* alleles was detected by PCR after isolated colony selection. CFUs were found to be non-excised (f/f), excised at one allele (F/Δ), or excised at both alleles (Δ/Δ).
(**h**) Excision of *Notch1* alleles detected by PCR after isolated CFU colony selection.

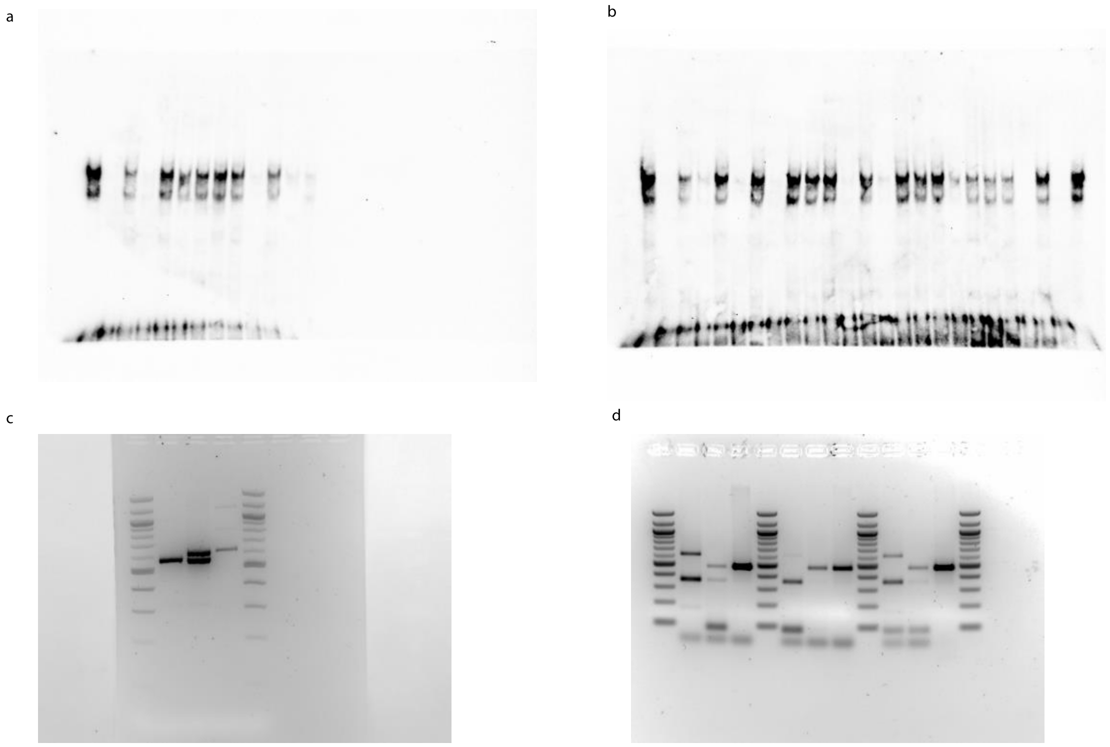

Supplementary Figure 6

a) EMSA gel from Figure 3b

b) EMSA gel from Figure 4b

c) Genotyping gel from Supplementary Figure 5g

d) Genotyping gel from Supplementary Figure 5g (left-most section)

Supplementary Table 1: Oligonucleotides used for RT-PCR expression analysis experiments

Target	Species	Forward primer	Reverse primer
Kit	Mouse	5’- AAATCCAGGCCCACACTCTG	5’-TAAGGAAGTTGCGTCGGGTC
Pecam1	Mouse	5’- GAGCCCAATCACGTTTCAGT	5’- TAAGGAAGTTGCGTCGGGTC
COUP-TFII	H/M	5’- CGGAGGAACCTGAGCTAC	5’- CCACTTTGAGGCACTTTTTGA
DLL4	Human	5’- GCCTATCTGTCTTTCGGGCT	5’- ATTGTGGGGGATGCATTCGT
	Mouse	5’- CCGGACTTTCTTCCGCATCT	5’- TGCCGCTATTCTTGTCCCTG
EFNB2	Human	5’- CCATGGTAACCAGCCACAGT	5’- CCCCTCTCCCCCATCCTAAA
	Mouse	5’- CGAGGTGGCAACAACAATGG	5’- ATAGTCCCCGCTGACCTTCT
EPHB4	Human	5’- GCGGAGTATCGGCGTCC	5’- AGCAGGGTCTCTTCCAAAGC
	Mouse	5’- GCACTTTGAACAGAGGGGGT	5’- GAGAGAGCCCTCTGGGAAGA
GAPDH	Human	5’- CCACTCCTCCACCTTTGA	5’- ACCCTGTTGCTGTAGCCA
	Mouse	5’- TGTGTCCGTCGTGGATCTGA	5’- CCTGCTTCACCACCTTCTTGA
Gata2	Mouse	5’- TCCAGCTTCACCCCTAAGCA	5’- ACAGGCATTGCACAGGTAGT
Hes1	Mouse	5’- CATGGAGAAGAGGCGAAGGG	5’- GGAATGCCGGGAGCTATCTTT
LEF1	H/M	5’- ATCTTCGCCGAGATCAGTCA	5’- GTTCTCTGGCCTTGTCGTGG
NOTCH1	Human	5’- GTTCTTGCAGGGGGTGC	5’- GGTGAGACCTGCCTGAATG
	Mouse	5’- AACAGTGCCGAATGTGAGTGG	5’- AAGTGACGCAAGAGCACCTAG
RUNX1	Human	5’- TTGGGGAGTCCCAGAGGTATC	5’- CGGAGCGAAAACCAAGACAG
	Mouse	5’- GACCGCAGCATGGTGGAGGT	5’- GTCTTGTTGCAGCGCCAGTG
SOX7	Human	5’- CTCTCCTGGGACAGCGTCA	5’- GCCAAGGACGAGAGGAAA
	Mouse	5’- GGGTCTCTTCTGGGACAGTG	5’- GGATGAGAGGAAACGTCTGG
SOX17	Human	5’- AGTGACGACCAGAGCCAGAC	5’- CCTTAGCCCACACCATGAAA
Sox17	Mouse	5’- CAGTAAGCCAGATTTGGTCTCTGA	5’- CCAAGACCTCTTGGGGAAATAGG
***Sox17* ORF**	Mouse	5’- AAAGACGAACGCAAGCGGTT	5’- GTCAACGCCTTCCAAGACTT
Sox18	Mouse	5’- TTGTAGTTGGGATGGTCGC	5’- CGCAGTACTGAGCAAGATGC

Supplementary Table 2: *Cdh5*(PAC)-CreERT2/R26RTd/*Sox17^{flox}* explant statistics

**Sox17** Experiment	**Genotype**	**Mean**	**SEM**	**N**	**Student's t-test** *p*-value	**2-way ANOVA *p*-value[1]** Column factor	Row Factor	**Figure**
**HE Ratio**	f/+	0.5187	0.04758	45	***$p<0.0001$	*** $p<0.0001$	*** $p<0.0001$	2d
	f/f	1.701	0.1645	38				
**BrdU EC**	f/+	3.755	0.5285	14	n.s.	n.s.	n.s.	Supp 2d
	f/f	2.999	0.5819	15				
**BrdU HC**	f/+	35.28	9.048	14	n.s.	n.s.	n.s.	Supp 2d
	f/f	52.58	9.344	15				
**Annexin-V EC**	f/+	54.54	3.958	18	n.s.	n.s.	n.s.	Supp 2d
	f/f	54.62	3.072	13				
**Annexin-V HC**	f/+	55.67	4.624	16	n.s.	n.s.	n.s.	Supp 2d
	f/f	55.53	2.915	13				
**CD41$^+$ %Td$^+$**	f/+	13.03	1.209	37	** $p<0.0085$	*** $p<0.0001$	*** $p<0.0001$	2e
	f/f	18.28	1.532	26				
**CD31$^+$117$^+$Sca1$^+$CD45$^+$ %Td$^+$**	f/+	11.8	2.678	14	*** $p<0.0006$	n.s.	*** $p<0.0030$	2f
	f/f	35.91	4.423	27				
**CD31$^-$117$^+$Sca1$^+$CD45$^+$ %Td$^+$**	f/+	6.399	2.683	16	** $p<0.0062$	n.s.	* $p<0.0189$	Supp 2e
	f/f	22.07	3.906	24				
**HE Ratio (+NICD)**	f/+ (-NICD)	1.079	0.1638	7				5b
	f/+ (+NICD)	1.370	0.1263	3		* $p<0.205$	** $p<0.0023$	
	f/f (-NICD)	1.998	0.2614	5	** $p<0.0010$			
	f/f (+NICD)	1.131	0.1192	8				
**Total cells %CD31$^+$**	f/+	13.23	1.287	13	n.s.	n.s.	n.s.	Supp 2a
	f/f	12.04	1.214	16				
**Total cells %CD45$^+$**	f/+	2.962	0.2206	15	n.s.	n.s.	n.s.	Supp 2a
	f/f	3.306	0.2622	21				
**%ECs Td$^+$**	f/+	17.84	1.909	10	n.s.	n.s.	n.s.	Supp 2a
	f/f	16.7	1.913	10				
**Total cells %Td$^+$**	f/+	3.577	0.3987	22	n.s.	n.s.	n.s.	Supp 2a
	f/f	3.92	0.5669	22				
**E9.5 HE Ratio**	f/+	2.448	0.6427	12	n.s.	n.s.	** p<0.0018	Supp 2f
	f/f	4.426	0.5841	10				

[1]For 2-way ANOVA, each litter is treated as a different group.

Supplementary Table 3: Oligonucleotides used in quantitative PCR for SOX17 and RUNX1 ChIP

Species (IP)	5'UTR Region	Site	ATG Position	Forward primer	Reverse primer
Mouse (SOX17)	***Lef1***			5'- CCTCCAGCAGATTAAATGCT	5'- GGAGGTACGAGAAGAATCAG
	CoupTFII	A	-17 bp to -284 bp	5'- TCCGGACTTCTGCTCCCCT	5'- ACAAACACACCGGGCCAGACA
		B	-327 bp to -451 bp	5'- AGAGAGTGGGAGCAGAACGT	5'- CGAGCGAGATCTTTAGAGAG
		C	-2420 bp to -2613 bp	5'- CACCCTCTGTACACACATGT	5'- CTCTTATGAGTTATGCTGGT
	Dll4	A	-26 bp to -240 bp	5'- CGCTCGAGACCCTAGGATTT	5'- GGACTCCGAATCTGCTTGTT
		B	-271 bp to -448 bp	5'- TGCTGGGACTGTAGCCACTA	5'- ACTTTGGCTGCAGCTCTTGG
		C	-1603 bp to -1803 bp	5'- AATTCTCCATCACCACCACC	5'- CTGTGGCTTCAGCTGTCA
	Gata2	A	-284 bp to -523 bp	5'- AGGAACTGCGGGTGCGTTTT	5'- TAGGTCCTGACATCGGTGAC
		B	-882 bp to -1162 bp	5'- AGAGGTTGGAAGACCTGAGC	5'- ACTCCTGCACAGACGTGAAG
		C	-1327 bp to -1540	5'- TTCAGCCTGGTGGTCTACTA	5'- CTCTCTGTCCTTCTATCAGG
	Notch1	A	-343 bp to -454 bp	5'- CTGGTTCCTGCGAACCCTT	5'- GATCCTTAGATCCTGGCTC
		B	-497 bp to -640 bp	5'- GGGCATCTAGGAACTACTTC	5'- CCGTACCTCCTCTACTATTG
		C	-1024 bp to -1172 bp	5'- TTCCACGGTCACCCTTCTCA	5'- GCTTAGCACAGGATGTCCA
		D	-1152 bp to -1357 bp	5'- TTGGACATCCTGTGCTAAGC	5'- TGCCTTTCAGGAACAGGTGT
		E	-1566 bp to -1775 bp	5'- CTGAAGGCCTCTAACTGCTT	5'- TCTGCTGTGCAGCCATACTC
	Runx1	A	-180 bp to -396 bp	5'- GTGGGGGAAAGAATTATTGC	5'- AGAACCACAAGTTGGGTAGC
		B	-572 bp to -697 bp	5'- CCAGGCTGTGTAAGGAAACA	5'- ACAGGACAGAGAGAGCAAGA
Mouse (RUNX1)	***Sox17***	A	-76 bp to -202 bp	5'- AGTGTCACTAGGCCGGCT	5'- GGAGTGAGGCACTGAGATGC
		B	-184 bp to -322 bp	5'- TGGGACGTGGGACTCGGA	5'- AGCCGGCCTAGTGACACT
		C	-304 bp to -405 bp	5'- AGCTCCGGCTAGTTTTCCCG	5'- TCCGAGTCCCACGTCCCA
		D	-1081 bp to -1277 bp	5'- TTTGCTATTGCTGGAGGGCG	5'- GCGGTTATTCTGGCAGAT
Human (SOX17)	***COUP-TFII***	A	-55 bp to -142 bp	5'- CTGCAGGCTAGTGCCTACTT	5'- TGTTGGCCCCCTGAAAAGAT
		B	-237 bp to -323 bp	5'- AAGGCGAGGCTTGCATTCCT	5'- GTATTAGGCTCTCTCAGC
		C	-1161 bp to -1420 bp	5'- GGAAAAACTTCTGTAGCCC	5'- ATGGAACTAACGCTCTTCGG
	DLL4	A	-317 bp to -445 bp	5'- TGGGACTGTAGCAGCTAGAGG	5'- AGCGCCGCTACTGAAACC
		B	-727 bp to -875 bp	5'- TGGGCACTCATAGGTTGG	5'- AGGCGCTAGTTACCTAGTGT
	GATA2	A	-29 bp to -171 bp	5'- CTTCTCCAGTCCTCAGAGAA	5'- CCCAAAACACCTTTAGAGGG
		B	-254 bp to -443 bp	5'- AGATTCTGGGGGCTGCGTTGA	5'- AAATGCGACGCCAAGTAGCA
		C	-423 bp to -595 bp	5'- TGCTACTTGGCGTCGCATTT	5'- AGTTCTGCCAGGTCCTTTCA
		D	-1247 bp to -1337 bp	5'- CGTAAGCTAAAGGATGGGA	5'- TTGGCGTTCCCCTCAACGC
		E	-1513 bp to -1642 bp	5'- GGGAGGCTTAGCAGGCGGCT	5'- TTTGTCTGTCCGAGGCCTCA
	NOTCH1	A	-262 bp to -401 bp	5'- TGTTGCAGGCCTCGTCCTTT	5'- CTGCCTCACACACAGAGAGT
		B	-581 bp to -763 bp	5'- TACCCTCCCTGGACCCAGTT	5'- GACCCTATCCCATGCCTCAT
		C	-703 bp to -867 bp	5'- TGTAGGCCTCGAGAGCTGCA	5'- CTGAGCCACGTGGAAAGG
	RUNX1	A	-1 bp to -263 bp	5'- CGCTTCCTCCTGAAAATGCA	5'- CATCACCAACCCACAGCCAA
		B	-263 bp to -449 bp	5'- TTGGTGTGGGTTGGTGATG	5'- CTGTGGAAAGGGGAACAGTT
		C	-567 bp to -675 bp	5'- ATAGCCGAGTAGACTTTGC	5'- TAACAACAGGAGCCGAGTTG
		D	-1088 bp to -1354 bp	5'- CACACACACACACACACACA	5'- AAGTGTCTCCTCCTGGTTC
		E	-1406 bp to -1603 bp	5'- GACATGCCTGTTTGAAGATG	5'- TAGGCAGAGCAGAGCCAAAT

Supplementary Table 4: Duplex oligonucleotides used in EMSA

ChIP site	Sox17 binding seq	EMSA probe sequence (duplex)
**Lef1**		WT 5'- CATTTCTTTATGTCCTTTGTTTACTGTTCTG (-3'BIOTIN)
		MT 5'- CATTTCTTTATGTC*AGGGTGG*TACTGTTCTG
CoupTFII site A	**COUPTF2_A1**	WT 5'- CGCGCCGCCTTTTGTGTGTGC
		MT 5'- CGCGCCG*GGGTGG*GTGTGTGC
	**COUPTF2_A2**	WT 5'- TTTTGCAAAGTTTTGTCGATTG
		MT 5'- TTTTGCAAAG*GGGTGG*CGATTG
CoupTFII site B	**COUPTF2_B**	WT 5'- GCTGCAAGTCGATTGTCTGGC
		MT 5'- GCTGCAAGTC*GGGTGG*CTGGC
CoupTFII site C	**COUPTF2_C**	WT 5'- ACTTCACCTCATTGTTATGATG
		MT 5'- ACTTCACCT*GGGTGG*TATGATG
Dll4 site C	**Dll4_C1**	WT 5'- AGAGGAATATTGTAATAGGT
		MT 5'- *TGGGGGACCCAGAGAGAAGG*
	**Dll4_C2**	WT 5'- GCACTGATCTTATCGTCCGACCAT
		MT 5'- GCACTGAT*AGGGTG*GTCCGACCAT
Gata2 site B	**Gata2_B1**	WT 5'- GCGCGGCGCTGATTGGCTGG
		MT 5'- GCGCGGCGCTG*GG*TGGCTGG
	**Gata2_B2**	WT 5'- CGGGAGCAGCCAATGGGGGG
		MT 5'- CGGGAGCAG*GGGTG*GGGGGG
Gata2 site C	**Gata2_C**	WT 5'- CGCGACCATTATTGGTCTAGC
		MT 5'- CGCGACC*GGGTGG*GGTCTAGC
Notch1 site A	**Notch1_A1**	WT 5'- CCCTTACCCCCTTGTGGACCC
		MT 5'- CCCTTACCC*AGGGTG*GGACCC
	**Notch1_A2**	WT 5'- TCGCAAGACAAGGAGGAATGG
		MT 5'- TCGCAAGA*AGGGTG*GGAATGG
	**Notch1_A3**	WT 5'- GTCGACTATATTCCAGCTTTGTCAGCA
		MT 5'- GTC*AAAGGGCCCCCACCCGGGAAA*GCA
Notch1 site B	**Notch1_B**	WT 5'- GGATCAGGCTTTGTGTGTAGCCGC
		MT 5'- GGATCAGG*AGGGTGG*TGTAGCCGC
Notch1 site C	**Notch1_C**	WT 5'- GTTCCAGTACAATGACTGCTAGCG
		MT 5'- GTTCCAGTA*AGGGTGG*TGCTAGCG
Notch1 site D	**Notch1_D**	WT 5'- CCAGATTATATTGTCCTAGGACCC
		MT 5'- *GGGTCCTAGGACAATATAATCTGG*
Notch1 site E	**Notch1_E1**	WT 5'- TCCACTGTCTTTGTCTAGCAATGA
		MT 5'- TCCACTGT*AGGGTGG*TAGCAATGA
	**Notch1_E2**	WT 5'- TGTGTCGACTATATTCCAGCT
		MT 5'- TGTGTCGA*AGGGTGG*CCAGCT
Runx1 site A	**Runx1_A1**	WT 5'- AAATATTCAAATTGTTAAAGATTA
		MT 5'- AAATATTCAAA*GGGGA*AAAGATTA
	**Runx1_A2**	WT 5'- GTTTGCATTCAGTGTGATTCGTCC
		MT 5'- GTTTGCAT*AGGGTGG*GATTCGTCC

Supplementary Table 5: *Cdh5*(PAC)-CreERT2/R26RTd/*Notch1*^{flox} and WT explant statistics

**Notch1** Experiment	**Genotype**	**Mean**	**SEM**	**N**	**Student's t-test** *p*-value	**2-way ANOVA *p*-value[1]** Column factor	Row factor	**Figure**
**HE Ratio**	f/+	0.8455	0.08111	18	* $p<0.038$	* $p<0.0107$	*** $p<0.0001$	4d
	f/f	1.893	0.2834	21				
**BrdU EC**	f/+	4.897	1.33	6	n.s.	n.s.	n.s.	4g
	f/f	5.527	0.7001	10				
**BrdU HC**	f/+	42.57	3.258	6	* $p<0.018$	n.s.	* $p<0.0494$	4g
	f/f	64.9	5.497	10				
**Annexin-V EC**	f/+	54.60	1.852	7	n.s.	n.s.	n.s.	Supp 4f
	f/f	55.17	2.40	6				
**Annexin-V HC**	f/+	48.09	3.435	7	n.s.	n.s.	n.s.	Supp 4g
	f/f	45.20	2.936	6				
**CD41$^+$ %Td$^+$**	f/+	7.26	1.552	12	** $p<0.0020$	*** $p<0.0001$	*** $p<0.0007$	4e
	f/f	12.83	1.994	13				
**CD31$^+$117$^+$Sca1$^+$CD45$^+$ %Td$^+$**	f/+	5.101	1.499	10	** $p<0.0084$	** $p<0.0027$	*** $p<0.0002$	4f
	f/f	15.21	3.513	6				
**CD31$^-$117$^+$Sca1$^+$CD45$^+$ %Td$^+$**	f/+	14.33	6.925	10	n.s.	n.s.	n.s.	Supp 4d
	f/f	17.81	9.02	6				
**Total cells %CD31$^+$**	f/+	13.18	0.758	14	n.s.	n.s.	n.s.	Supp 4h
	f/f	12.22	0.7345	8				
**Total cells %CD45$^+$**	f/+	1.78	0.2435	15	n.s.	n.s.	n.s.	Supp 4h
	f/f	2.027	0.2379	7				
**%ECs Td$^+$**	f/+	17.67	3.196	6	n.s.	n.s.	n.s.	Supp 4h
	f/f	19.58	2.075	13				
**Total cells %Td$^+$**	f/+	2.761	0.7531	9	n.s.	n.s.	n.s.	Supp 4h
	f/f	4.366	0.761	9				
**Wildtype HE ratio (DAPT)**	WT +DMSO	0.7825	0.05456	7	control	**1-way ANOVA *p*-value**		Supp 4e
	WT +25μM	0.9771	0.07913	4	n.s.	** $p<0.058$		
	WT +50μM	0.9702	0.02999	9	** $p<0.001$			
	WT +100μM	0.9649	0.01957	8	** $p<0.001$			
	WT +200μM	0.8264	0.0321	3	n.s.			

[1]For 2-way ANOVA, each litter is treated as a different group.

Supplementary Table 6: Oligonucleotides used for mouse genotyping and excision PCR

Line		Forward primer	Reverse primer
***Cdh5*(PAC)-CreERT2**	+/-	5'- ATCCAGGTTACGGATATAGT	5'- CCAAAATTTGCCTGCATTACCGGTCGA
**R26R-tdTomato**	WT/Td	5'- CTCTGCTGCCTCCTGGCTTCT	5'- CCAGGCGGATCACAAGCAATA
			5'- TCAATGGGCGGGGGTCGTT
Notch1 $^{f/f}$	WT/Flox	5'- CTGACTTAGTAGGGGGAAAAC	5'- AGTGGTCCAGGGTGTGAGTGT
	Δ		5'- TAAAAAGCGACAGCTGCGGAG
**R26R-NICD-GFP**	+/-	5'- ACAGATCTGGATGCCCGAAT	5'- TTGTTGGCTCCGTTCTTCAG
Sox17 $^{f/f}$	WT/Flox	5'- TTGCCGAACACACAAAAGGAG	5'- TGGAGGTGCTGCTCACTGTAA
Sox17 $^{f/f}$ **excision**	WT/Flox	5'- TCTTGATCCCACTTCCCACA	5'- GGACTGGAAAATGAGAGAATA
	Δ	5'- TTGCCGAACACACAAAAGGAG	5'- GGACTGGAAAATGAGAGAATA
**TP1-Venus (ICR)**	+/-	5'- GGCAGATCACTTCAGCTTCTGC	5'- CGTTCTTCTGCTTGTCGGCGG
**Mlc2a** $^{f/f}$	Cre	5'- GGCACGATCACTCAGTCAGA	5'- CCTGTTTTGCACGTTCACCG
	WT/Flox		5'- ATCCCTGTTCTGGTCAATGC

Supplementary Table 7: Antibodies used for ChIP, IF, and Flow Cytometry

Antibody	Target	Manufacturer	Clone / Catalog #	Working Concentration	Source/Isotype
1° Ab	***CD117***	BD Pharmingen	553352	1:100	Rat
	CD144	Novus	NBP1-43348	5μg/mL	Rat
	CD31	BD Pharmingen	553370	1:100	Rat
	CD41	Abcam	Ab11024	1:100	Rat
	CD45	Abcam	Ab10558	1:100	Rabbit
	Gata2	Pierce	PA1-100	1:100	Rabbit
	Runx1	Abcam	Ab92336	1:100	Rabbit (monoclonal)
	Runx1*	Abcam	Ab35962	1:100	Rabbit (polyclonal)
	***Runx1* (AML1 ChIP)**	Cell Signaling	D4A6	1:25	Rabbit (monoclonal)
	Sox17	R&D Systems	AF1924	4μg/mL (IF), 8μg/mL (ChIP)	Goat
2° Ab	**Alexa 488 α-goat**	Invitrogen	A11055	1:100	Donkey
	**Alexa 488 α-rabbit**	Invitrogen	A21206	1:100	Donkey
	**Alexa 488 α-rat**	Invitrogen	A21208	1:100	Donkey
	**Alexa 594 α-mouse**	Invitrogen	A21203	1:100	Donkey
	**Alexa 594 α-rabbit**	Invitrogen	A21207	1:100	Donkey
	**Alexa 594 α-rat**	Invitrogen	A21209	1:100	Donkey
	**Alexa 647 α-goat**	Invitrogen	A21447	1:100	Donkey
	**Alexa 647 α-rabbit**	Invitrogen	A31573	1:100	Donkey
Conjugated	**CD117-APC**	BD Biosciences	553356	1:100	Rat IgG2b, κ
	**CD31-APC**	BD Biosciences	551262	1:100	Rat IgG2a, κ
	**CD31-PE**	BD Biosciences	553373	1:100	Rat IgG2a, κ
	**CD4-PB**	Invitrogen	MCD0428	1:100	Rat IgG2a, κ
	**CD41-FITC**	BD Biosciences	553848	1:100	Rat IgG1, κ
	**CD45-FITC**	BD Biosciences	553080	1:100	Rat IgG2b, κ
	**CD45-PerCP**	Biolegend	103130	1:100	Rat IgG2b, κ
	**CD8-FITC**	BD Biosciences	553031	1:100	Rat IgG2a, κ
	**Ly6A/E (Sca1)-PE-Cy7**	BD Biosciences	558162	1:100	Rat IgG2a, κ
	**TCRβ-APC**	Biolegend	109212	1:100	Armenian Hamster IgG
Isotype controls	**IgG-APC**	Biolegend	400912	1:100	Armenian Hamster IgG
	**IgG-APC**	BD Biosciences	553991	1:100	Rat IgG2b, κ
	**IgG-APC**	BD Biosciences	554690	1:100	Rat IgG2a, κ
	**IgG-FITC**	BD Biosciences	554684	1:100	Rat IgG1, κ
	**IgG-FITC**	BD Biosciences	554688	1:100	Rat IgG2a, κ
	**IgG-FITC**	BD Biosciences	556923	1:100	Rat IgG2b, κ
	**IgG-PE**	BD Biosciences	554689	1:100	Rat IgG2a, κ
	**IgG-PE-Cy7**	BD Biosciences	552784	1:100	Rat IgG2a, κ
	**IgG-PerCP**	Biolegend	400336	1:100	Rat IgG2a, κ
	**IgG (AML1 ChIP)**	Cell Signaling	2729S	1:25	Rabbit
	**IgG (Sox17 ChIP)**	Santa Cruz Biot	SC-3887	1:12.5	Goat
Other	**AnnexinV-FITC**	BD Pharmingen	556570	1:20	
	**BrdU Cell Cycle**	BD Pharmingen	558662	kit (as directed)	
	**DAPI**	Sigma Aldrich	D9542	80pg/mL (IF), 20pg/mL (FACS)	
	**Lectin HPA Alexa-488**	Life Technologies	L11271	1:100	Helix pomatia snail

* Used as a confirmatory antibody for Runx1 monoclonal antibody.

Supplementary Methods

Scanning electron microscopy

Embryos were fixed in 4% glutaraldehyde/ 4% EM grade formaldehyde then PBS washed and mounted in 4% low melting point agarose and sectioned from 50-200 µm. Tissues were collected and washed in PO4 for 15 min followed by 1% OsO4 (dH2O) for 1hr room temperature, then washed in dH20 and dehydrated following stepwise increase from 35% to 95% EtOH followed by three washes in 100% EtOH. Slides were then transferred to a critical point dryer and samples mounted on aluminum stubs. Tissues were coated with palladium:gold sputter coat under high vacuum prior to evaluation with a Carl Zeiss Ultra 55 Field Emission Scanning Electron Microscope (Zeiss).

Hematopoietic assays

Td^{+}/CD117-APC^{+}/CD45-$FITC^{+}$ DAPI-excluded cells from dissected AGMs of *in vivo* tamoxifen induced embryos were sorted into IMDM 2% FBS collection medium. For methylcellulose colony formation assay, cells were combined with Methocult medium (Stem Cell Technologies, M3434) supplemented with 10% IMDM/FBS and plated at 90-100 cells/mL. Colonies were scored at 1 week and picked for excision genotyping (see Supplementary Table 6 for list of primers). OP9-DL1 T-lymphoid differentiation assays were performed in α-MEM (Invitrogen, 12561056) supplemented with 20% heat-inactivated FBS, 5 ng/mL recombinant human Flt-3L (R&D Systems, 308-FK), 1 ng/mL recombinant murine IL-7 (Peprotech, 217-17), and penicillin-streptomycin[1]. 300 cells from each AGM were sorted onto OP9-DL1 monolayers, and then mechanically passaged and filtered (Falcon, 352235) every 5-7 days for 5 weeks before flow cytometric analysis.

Luciferase reporter assay fragment sequences

Gata2 (724bp)

5’AGGCCCCGCCCGGAGCCCTTCCCCCTCCCTGGGCCACTGGCTTGACCGCGACCATTATTGGTCTAGCACAGCC
TCAAGTGTCTTAGTGCTCAAAGTTCGGGTGCCCTAGAGAAGTCCACAATCCCTAGACTCATGTTGTCCAGCGGAT
CCTACCAGCCTCTTGCACAGCTATCCCTGATAGAAGGACAGAGAGTTTGGGGAGTCAGTTGGATTTGGGCTGGCC
GTCGTCCGTAGCAGTGGAGGTGGGGCTCCGCCCGAGAGTAGAAAGCTGTGGTCCCAGCAGAGAGATACCCAGA
AGGTGCACGTCTCGGCTCCTGGGAAGTCAGGGACCCTATTCGTGCCTAGTTGCTGGGAGGGCAGAGGTTGGAAG
ACCTGAGCGTCTGCCGGAGGGGTGCAGGGTCTGCCCACGGCGAAGGTCCCCTGGGGGGGGGGGCGTTGGCAT
CAGAGGCCGCAGAGAGGGCGCTGGTAGGGGGCCAGGCAGCCTAGGAGGCCAGCTTGCGGGTCATTCCCGAAG
TCCAGCGGCCAAAGCGGCGGGAGCAGCCAATGGGGGGGCGGAGGCTGGGCGGCGCGCGGCGCTGATTGGCT
GGCGCCGGCTTCATAGGCGTGCGCGGCCCCCGCTTCACGTCTGTGCAGGAGTCGGCAGCTGGCGCCAGGGCG
GCCCGGAGGATGCAGAGGGGCCGGAGCCGGGCGGGCCGGAAGCCGAGACGCGCGCTGTCCCCCACCC

Runx1 (851bp)

5’GCATCCGGGCTCAGCAGCAAGTTGGTGCCAACGTTGAATTGCTGTTGAATAAACAGCAAGGCAATCTTTATCTA
AATAATCAGTTGTTCCTCAAACCACAAATAACAACAGGATCTGAAAGCCACCAAATCCGCACAGGACAGAGAGAG
CAAGAAAAGACTGAGGCAGGGGATTTCTGTTTGCTTGTTTGTTGTGCTTTTTTTTCTCTTACAGCCCCTCTCTGCTA
AGCTCTGCTCAACTGTTTCCTTACACAGCCTGGGGGAGGGCAGGTGGAGGGCAGGAAGGGCATAGCTCAGAAAG
TTTAAAAAAAAAAAATTGACATCACTTAAGTCACGTGATTGGCAAGAGCCAATGGCGGTGGGCTGTGGAAAGGGG
AACAGTTAAATTTGTAATTTGGGTTGTGTGAAAACTTCTTTGGACCTCATAAACAACCACAGAACCACAAGTTGGGT
AGCCTGGCAGTGTCAGAAGTGTAAGCCCAGCACAGTGGTCAGCAGGCAGGACGAATCACACTGAATGCAAACCA

CAGGCTTTCGCAGAGCGGTGAGCAGTTCAACCCACAGCATAGGCGGTGCTTTCGTCTTTTTTTTTTCCTTTTTTTA
ATCTTTAACAATTTGAATATTTGTTTCTGCAATAATTCTTTCCCCCACCCCCACCCCATAGGACCCATGGAGTACCA
GAAGTGTTAGGGTTGGGGGTAGAAAGAGACGTGGGGAGCCATGGTGGGAGGTGAGGTCAGAGTAAGTGACATTT
CTTGGTTTTTGCTCTGAAGGTGAAAGAAATTATAGAATCCCCCGCCTTCAGGAGAGGTGCGTTTTCGAAAGGAAAC
GATGGCTTCAGACAGCATTT

Supplementary References

1. Holmes, R. & Zuniga-Pflucker, J. C. The OP9-DL1 System: Generation of T-Lymphocytes from Embryonic or Hematopoietic Stem Cells In Vitro. *Cold Spring Harbor Protocols* (2009).
